# Insights into chemoautotrophic traits of a prevalent bacterial phylum CSP1-3, herein *Sysuimicrobiota*

**DOI:** 10.1093/nsr/nwae378

**Published:** 2024-10-23

**Authors:** Lan Liu, Zheng-Han Lian, Ai-Ping Lv, Nimaichand Salam, Jian-Chao Zhang, Meng-Meng Li, Wei-Min Sun, Sha Tan, Zhen-Hao Luo, Lei Gao, Yang Yuan, Yu-Zhen Ming, Yu-Ting OuYang, Yu-Xian Li, Ze-Tao Liu, Chao-Jian Hu, Ying Chen, Zheng-Shuang Hua, Wen-Sheng Shu, Brian P Hedlund, Wen-Jun Li, Jian-Yu Jiao

**Affiliations:** State Key Laboratory of Biocontrol, Guangdong Provincial Key Laboratory of Plant Stress Biology and Southern Marine Science and Engineering Guangdong Laboratory (Zhuhai), School of Life Sciences, Sun Yat-Sen University, Guangzhou 510275, China; State Key Laboratory of Biocontrol, Guangdong Provincial Key Laboratory of Plant Stress Biology and Southern Marine Science and Engineering Guangdong Laboratory (Zhuhai), School of Life Sciences, Sun Yat-Sen University, Guangzhou 510275, China; State Key Laboratory of Biocontrol, Guangdong Provincial Key Laboratory of Plant Stress Biology and Southern Marine Science and Engineering Guangdong Laboratory (Zhuhai), School of Life Sciences, Sun Yat-Sen University, Guangzhou 510275, China; National Agri-Food Biotechnology Institute, Mohali 140306, India; School of Earth System Science, Institute of Surface-Earth System Science, Tianjin University, Tianjin 300072, China; State Key Laboratory of Biocontrol, Guangdong Provincial Key Laboratory of Plant Stress Biology and Southern Marine Science and Engineering Guangdong Laboratory (Zhuhai), School of Life Sciences, Sun Yat-Sen University, Guangzhou 510275, China; National-Regional Joint Engineering Research Center for Soil Pollution Control and Remediation in South China, Guangdong Key Laboratory of Integrated Agro-Environmental Pollution Control and Management, Institute of Eco-Environmental and Soil Sciences, Guangdong Academy of Sciences, Guangzhou 510650, China; State Key Laboratory of Biocontrol, Guangdong Provincial Key Laboratory of Plant Stress Biology and Southern Marine Science and Engineering Guangdong Laboratory (Zhuhai), School of Life Sciences, Sun Yat-Sen University, Guangzhou 510275, China; State Key Laboratory of Biocontrol, Guangdong Provincial Key Laboratory of Plant Stress Biology and Southern Marine Science and Engineering Guangdong Laboratory (Zhuhai), School of Life Sciences, Sun Yat-Sen University, Guangzhou 510275, China; State Key Laboratory of Desert and Oasis Ecology, Key Laboratory of Ecological Safety and Sustainable Development in Arid Lands, Xinjiang Institute of Ecology and Geography, Chinese Academy of Sciences, Urumqi 830011, China; State Key Laboratory of Biocontrol, Guangdong Provincial Key Laboratory of Plant Stress Biology and Southern Marine Science and Engineering Guangdong Laboratory (Zhuhai), School of Life Sciences, Sun Yat-Sen University, Guangzhou 510275, China; State Key Laboratory of Biocontrol, Guangdong Provincial Key Laboratory of Plant Stress Biology and Southern Marine Science and Engineering Guangdong Laboratory (Zhuhai), School of Life Sciences, Sun Yat-Sen University, Guangzhou 510275, China; State Key Laboratory of Biocontrol, Guangdong Provincial Key Laboratory of Plant Stress Biology and Southern Marine Science and Engineering Guangdong Laboratory (Zhuhai), School of Life Sciences, Sun Yat-Sen University, Guangzhou 510275, China; Chinese Academy of Sciences Key Laboratory of Urban Pollutant Conversion, Department of Environmental Science and Engineering, University of Science and Technology of China, Hefei 230026, China; State Key Laboratory of Biocontrol, Guangdong Provincial Key Laboratory of Plant Stress Biology and Southern Marine Science and Engineering Guangdong Laboratory (Zhuhai), School of Life Sciences, Sun Yat-Sen University, Guangzhou 510275, China; State Key Laboratory of Biocontrol, Guangdong Provincial Key Laboratory of Plant Stress Biology and Southern Marine Science and Engineering Guangdong Laboratory (Zhuhai), School of Life Sciences, Sun Yat-Sen University, Guangzhou 510275, China; State Key Laboratory of Biocontrol, Guangdong Provincial Key Laboratory of Plant Stress Biology and Southern Marine Science and Engineering Guangdong Laboratory (Zhuhai), School of Life Sciences, Sun Yat-Sen University, Guangzhou 510275, China; Chinese Academy of Sciences Key Laboratory of Urban Pollutant Conversion, Department of Environmental Science and Engineering, University of Science and Technology of China, Hefei 230026, China; Institute of Ecological Science, Guangzhou Key Laboratory of Subtropical Biodiversity and Biomonitoring, Guangdong Provincial Key Laboratory of Biotechnology for Plant Development, School of Life Sciences, South China Normal University, Guangzhou 510631, China; Guangdong Provincial Key Laboratory of Chemical Pollution, South China Normal University, Guangzhou 510006, China; School of Life Sciences, University of Nevada Las Vegas, Las Vegas, NV 89154, USA; Nevada Institute of Personalized Medicine, University of Nevada Las Vegas, Las Vegas, NV 89154, USA; State Key Laboratory of Biocontrol, Guangdong Provincial Key Laboratory of Plant Stress Biology and Southern Marine Science and Engineering Guangdong Laboratory (Zhuhai), School of Life Sciences, Sun Yat-Sen University, Guangzhou 510275, China; State Key Laboratory of Desert and Oasis Ecology, Key Laboratory of Ecological Safety and Sustainable Development in Arid Lands, Xinjiang Institute of Ecology and Geography, Chinese Academy of Sciences, Urumqi 830011, China; State Key Laboratory of Biocontrol, Guangdong Provincial Key Laboratory of Plant Stress Biology and Southern Marine Science and Engineering Guangdong Laboratory (Zhuhai), School of Life Sciences, Sun Yat-Sen University, Guangzhou 510275, China

**Keywords:** Sysuimicrobiota phy. nov, facultative anaerobes, chemoautotrophic metabolisms

## Abstract

Candidate bacterial phylum CSP1-3 has not been cultivated and is poorly understood. Here, we analyzed 112 CSP1-3 metagenome-assembled genomes and showed they are likely facultative anaerobes, with 3 of 5 families encoding autotrophy through the reductive glycine pathway (RGP), Wood–Ljungdahl pathway (WLP) or Calvin-Benson-Bassham (CBB), with hydrogen or sulfide as electron donors. Chemoautotrophic enrichments from hot spring sediments and fluorescence *in situ* hybridization revealed enrichment of six CSP1-3 genera, and both transcribed genes and DNA-stable isotope probing were consistent with proposed chemoautotrophic metabolisms. Ancestral state reconstructions showed that the ancestors of phylum CSP1-3 may have been acetogens that were autotrophic via the RGP, whereas the WLP and CBB were acquired by horizontal gene transfer. Our results reveal that CSP1-3 is a widely distributed phylum with the potential to contribute to the cycling of carbon, sulfur and nitrogen. The name *Sysuimicrobiota* phy. nov. is proposed.

## INTRODUCTION

The Earth harbors a huge biodiversity of eukaryotic and prokaryotic microorganisms, and as many as 10^11^–10^12^ microbial species are estimated to inhabit Earth [1], with the number of bacterial and archaeal species exceeding 4 million on the basis of 16S rRNA gene sequence identities [2]. Unlike visible plants and animals, for which taxonomic research can be conveniently conducted, and relatively comprehensive and in-depth knowledge has been attained, microorganisms are invisible. Only a small portion of bacteria detected in the natural environment is cultivatable under laboratory conditions [3,4] and most bacterial phyla have no cultured members. With advances in high-throughput sequencing, single-cell sorting and bioinformatics, insight into ‘microbial dark matter’ [5] prokaryotes has become possible, and has enabled reconstruction of nearly complete or even closed genomes for uncultured prokaryotes directly from the environment, which has led to the discovery of many candidate phyla and dramatically expanded the tree of life [6–8]. All in all, there are still a large number of uncultivated microorganisms in the environment waiting to be excavated and analyzed.

The candidate bacterial phylum CSP1-3, as one of the uncultivated phyla, was initially discovered through 16S rRNA gene amplicon surveys and defined as the GAL15 group in the SILVA database. Subsequently, a high-quality metagenome-assembled genome (MAG) assigned to CSP1-3 was identified as a prominent member of a terrestrial aquifer and suggested to belong to the *Armantimonadota* [9]. However, according to the classification in the Genome Taxonomy Database (GTDB), release v214, CSP1-3 is considered a distinct phylum. Up to this point, less than 30 CSP1-3 MAGs have been reconstructed from various environments, including sediments [9–11], soils [12–14], groundwater [15], freshwater [16], hot springs [17], hydrothermal vents [18] and high-temperature bioreactors [19]. It has been suggested that members of this lineage employ the Wood–Ljungdahl pathway (WLP) for carbon fixation [9] and carry out several steps of denitrification according to metagenomic investigations [9,10,19]. Recent studies have also indicated that CSP1-3 members express genes involved in peptide and amino acid assimilation [11] and encode enzymes for carboxydotrophy and sulfur oxidation [20]. Although these observations provide valuable insights into the CSP1-3 lineage, a comprehensive understanding of the physiology, ecology and evolution of the group has been lacking.

Here, we describe a systematic study of 112 CSP1-3 MAGs and the chemoautotrophic enrichment of 6 genera of CSP1-3 in the laboratory that were identified in the enrichment by fluorescence *in situ* hybridization (FISH). Gene expression patterns and DNA-stable isotope probing (DNA-SIP) results were both consistent with the proposed chemolithoautotrophic lifestyles. Our research significantly broadens the genomic representation of CSP1-3, demonstrates autotrophy potential, and presents the first comprehensive blueprint of the phylum, herein named *Sysuimicrobiota*.

## RESULTS

### Taxonomic profile of *Sysuimicrobiota*

**Figure 1.**
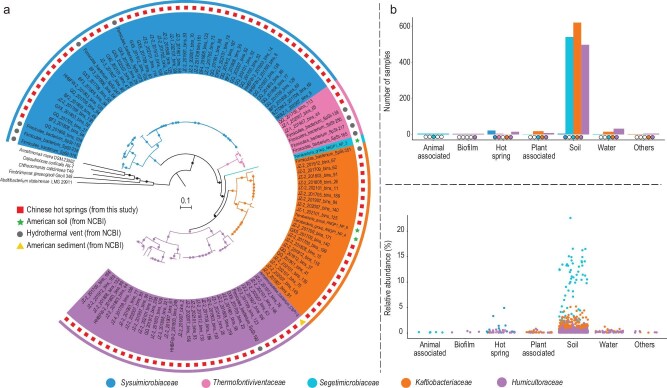
Phylogeny and geographic distribution of *Sysuimicrobiota*. (a) Phylogenetic placement of *Sysuimicrobiota* MAGs based on 31 concatenated bacterial marker genes. The IQ-Tree was used for phylogenetic inference [68], and the best-fit model LG + F + R4 was well supported by Bayesian Information Criterion (BIC). Bootstrap values were based on 1000 replicates and nodes with percentages >70% indicated as circles. (b) The distribution of *Sysuimicrobiota* based on IMNGS analysis of the target 16S rRNA genes. Top: the number of samples from different biomes containing the 16S rRNA gene to *Sysuimicrobiota*. Bottom: the relative abundance of each family of *Sysuimicrobiota* in different biomes; each dot represents a sample.

We retrieved 97 MAGs assigned to CSP1-3 from 39 metagenomes (Table S1) derived from hot spring sediments in Southwestern China and supplemented them with 15 MAGs from the National Center for Biotechnology Information (NCBI) database. Together, these genomes originated from various environments, such as hot springs (97 MAGs), hydrothermal vents (11 MAGs), temperate grassland soils (3 MAGs) and sediments from an alluvial aquifer (1 MAG) (Table S2). Phylogenetic analysis, employing Bac120 marker sets from GTDB, demonstrated that the genomes assigned to CSP1-3 form a monophyletic group within the ‘Terrabacteria’ (Fig. S1). Our analysis of the 16S rRNA gene phylogenetic tree further supported the designation of CSP1-3 as a phylum-level taxon in the domain *Bacteria* (Fig. S2). In order to resolve the phylogenetic affiliations of the MAGs within the CSP1-3, 5 type strains from 4 classes of *Armatimonadota* were used as the outgroup for phylogenomic analyses based on 31 universal marker proteins (Fig. 1a) [21] and 16 ribosomal proteins (Fig. S3) [6], and average amino acid identity (AAI) and average nucleotide identity (ANI) values were performed. The species delineations were performed using AAI (AAI: 95%–100% for same species) [22] and ANI (95%–100% for same species) [23] values (Figs S4 and S5), which were consistent with the GTDB classification [24] (Table S2). The delineation of higher taxonomic ranks was primarily conducted based on the phylogenomic tree and GTDB classification, as numerous studies have shown that using AAI and ANI to classify higher taxonomic ranks makes it difficult to establish consistent cut-off values. Finally, we propose new taxonomic names based on the SeqCode, and the CSP1-3 MAGs represent one phylum with the proposed name *Sysuimicrobiota*, along with one class, one order and 5 families: *Sysuimicrobiaceae, Thermofontiviventaceae, Segetimicrobiaceae, Kaftiobacteriaceae* and *Humicultoraceae*, which contain 11 genera and 25 species (SeqCode ID: 32823 and the URL is https://seqco.de/i:32823) (Supplementary Data; Table S3). This analysis radically expands the diversity of known members of this phylum and is the first taxonomic study of this group.

### *Sysuimicrobiota* are globally distributed and commonly found in soil and aquatic environments

We applied Integrated Microbial Next Generation Sequencing (IMNGS: https://www.imngs.org/) [25] to investigate the distribution of *Sysuimicrobiota*. Our analysis revealed that 1432 environmental samples contain *Sysuimicrobiota* with relative abundance exceeding 0.1% (Table S4), including animal-associated, biofilm, hot spring, plant-associated, soil, water and other samples (Fig. 1b). The highest relative abundance of *Sysuimicrobiota* was observed in soils, reaching 22.64%, followed by hot springs, reaching 4.94%. In soils, hot springs, water and plant-associated samples, multiple families tend to coexist, with *Segetimicrobiaceae* being the most abundant in soils. In biofilms, only members of *Humicultoraceae* were detected. The family *Humicultoraceae* exhibits the broadest distribution across habitat categories, followed by *Kaftiobacteriaceae* and *Segetimicrobiaceae*. The family *Thermofontiviventaceae* was only detected in hot springs (Table S4). Furthermore, a tool for interrogating public shotgun metagenome data sets, Sandpiper [26], also showed a global distribution and high relative abundance of *Sysuimicrobiota* (https://sandpiper.qut.edu.au/taxonomy/p_CSP1-3). In all, *Sysuimicrobiota* occupies a broad ecological niche and is found worldwide.

### Facultative anaerobes, adaptability to extreme environments, and motility

**Figure 2.**
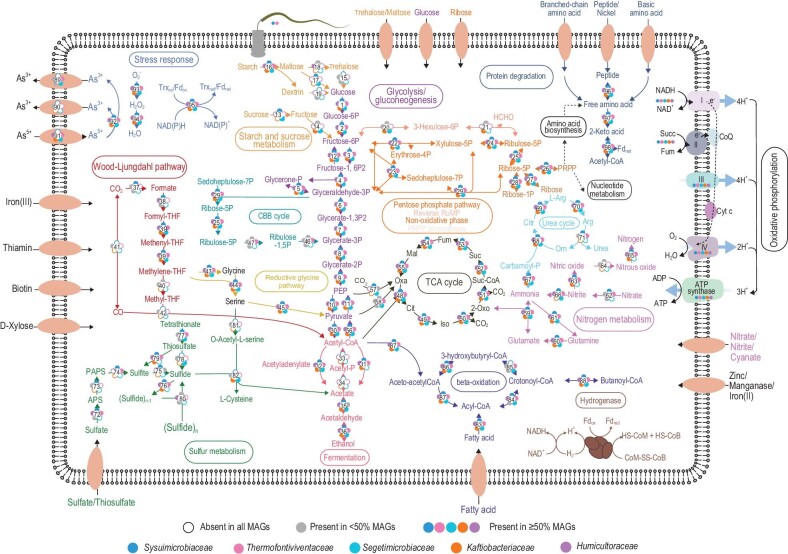
Overview of metabolic capabilities in five new families of *Sysuimicrobiota*. Genes involved in glycolysis, pentose phosphate pathway, TCA cycle, WLP, RGP, CBB, nitrogen metabolism, sulfur metabolism, membrane transporters and other functions are shown. The circles represent the different genes, and are colored by families of *Sysuimicrobiota*. The numbers inside the circle represent the genes presented in Table S5.

*Sysuimicrobiota* genomes contain genes for glycolysis, gluconeogenesis, phosphoribosyl pyrophosphate (PRPP) generation, non-oxidative pentose phosphate, tricarboxylic acid (TCA) cycle and *β*-oxidization pathways (Fig. 2, Fig. S6 and Table S5). Furthermore, all *Sysuimicrobiota* lineages contain complete pathways for aerobic respiration, acetyl-CoA synthetase (*acs*), aldehyde dehydrogenase (*aldh*) and alcohol dehydrogenases (*adh*), suggesting that *Sysuimicrobiota* are likely to be facultative anaerobes. Genes annotated to encode the chaperones GroEL, HSP70 (DnaK), HSP40 (DnaJ), the nucleotide exchange factor GrpE, the DNA repair protein RadA, thioredoxin reductase (TrxR), peroxiredoxins (PrxQ) and the metal efflux pumping ATPases (CopA and CopB) were prevalent, suggesting *Sysuimicrobiota* has mechanisms to cope with environmental stresses, such as heat [27], oxidative damage [28] and heavy metals [29]. A set of core genes [30] encoding bacterial flagellar components was identified in genomes of *Sysuimicrobiaceae* and *Thermofontiviventaceae* (Table S5), suggesting they may be motile. Moreover, genes encoding core chemotaxis signaling complexes [31] and methyl-accepting chemotaxis proteins were also detected in those genomes.

### Versatile carbon fixation pathways in *Sysuimicrobiota*

A previous study proposed that a single CSP1-3 MAG harbored genes for the WLP [9], which can convert two carbon dioxide molecules to acetyl-CoA and can operate in both the reductive (acetyl-CoA formation from CO_2_) and oxidative (acetyl-CoA degradation to two C1 compounds) modes [32]. We analyzed all 112 high-quality MAGs and discovered that only *Humicultoraceae* MAGs contain genes coding for the carbon monoxide dehydrogenase/acetyl-CoA synthase (CODH/ACS) complex. To explore the evolutionary history of the *Humicultoraceae* CODH/ACS complex (AcsA, AcsB, AcsC, AcsD and AcsE), we constructed phylogenetic trees from alignments of CODH/ACS subunits (Figs S7–S11), including sequences from *Actinomycetota* [33] and other bacteria [34]. Our phylogeny of CODH/ACS subunits is in overall agreement with two large clades corresponding to ‘Terrabacteria’ and ‘Gracilicutes’ [34]. The sequences from the *Humicultoraceae* MAGs formed a monophyletic group with other ‘Terrabacteria’ enzymes. The CODH/ACS complex was only present in one shallow-branching family (*Humicultoraceae*) of *Sysuimicrobiota*, and did not show a discrete distribution throughout *Sysuimicrobiota* with the absent in other clades within *Sysuimicrobiota*. Therefore, our findings strongly supported the possibility of a single acquisition event occurring in the lineage of *Humicultoraceae*, rather than the CODH/ACS complex being present in the last common ancestor of *Sysuimicrobiota* and subsequently lost in other *Sysuimicrobiota* clades. Interestingly, the genes for the methyl-branch of the WLP (*fdhA*, formate dehydrogenase; *fhs*, formate-tetrahydrofolate ligase; *folD*, methylenetetrahydrofolate dehydrogenase) were prevalent for *Sysuimicrobiota* MAGs (Fig. 2 and Table S5), which suggests that the *Sysuimicrobiota* may instead use the reductive glycine pathway (RGP) for CO_2_ assimilation.

The recently confirmed RGP [35–38] consists of the methyl-branch of the WLP plus the glycine cleavage system (GCS), which is composed of glycine dehydrogenase (GcvPA and GcvPB), aminomethyltransferase (GcvT), glycine cleavage system H protein (GcvH) and dihydrolipoyl dehydrogenase (PdhD). These core genes were present in 102 *Sysuimicrobiota* genomes assigned to all 5 families. Phylogenetic trees of key RGP proteins (GcvPA, GcvPB, GcvT, GcvH and PdhD) revealed they are monophyletic (Figs S12–S16). The wide phylogenetic distribution and monophyly of the RGP proteins suggests they were likely inherited vertically from the common ancestor of this phylum.

Besides the RGP pathway, MAGs assigned to the *Sysuimicrobiaceae, Thermofontiviventaceae, Kaftiobacteriaceae* and *Humicultoraceae* harbor some of the essential genes for the Calvin-Benson-Bassham (CBB), but most MAGs encoded incomplete pathways. Key genes identified include ribulose-1,5-bisphosphate carboxylase/oxygenase (RuBisCO) and phosphoribulokinase. A phylogeny of the RuBisCO large subunit, RbcL, revealed three different *Sysuimicrobiota* clades (Fig. S17). One of these clades, containing sequences from *Kaftiobacteriaceae* and *Humicultoraceae*, was related to the form IV group, which performs functions distinct from carbon fixation such as methionine salvage, sulfur metabolism and D-apiose catabolism [39], thus, we believe carbon fixation via the CBB is unlikely in *Kaftiobacteriaceae* and *Humicultoraceae*. However, sequences from *Sysuimicrobiaceae* and *Thermofontiviventaceae* comprise two monophyletic groups within the form I group, indicating that the *rbcL* gene was transferred to *Sysuimicrobiota* by at least two horizontal gene transfer (HGT) events. However, the RuBisCO small subunit, RbcS, was limited to three *Sysuimicrobiaceae* MAGs, indicating that the complete gene set for the CBB is present exclusively in members of *Sysuimicrobiaceae*.

Thus, overall, three autotrophic pathways are predicted with different distributions in the phylum *Sysuimicrobiota*. The CBB and RGP pathways are found within the *Sysuimicrobiaceae*. The RGP pathway is found in *Kaftiobacteriaceae*. And both WLP and RGP pathways are found within the *Humicultoraceae*.

### Energy conservation mechanisms in *Sysuimicrobiota*

The extensive genomic coverage in most *Sysuimicrobiota* MAGs allowed for a detailed investigation of potential energy conservation mechanisms. Five different types of hydrogenases (NiFe hydrogenase group 1f, 1g and 3b–3d) were identified in *Sysuimicrobiota* MAGs (Fig. S18), and the hydrogenases encoded by members of each family were distinct. Group NiFe 3b-type hydrogenases are present in *Sysuimicrobiaceae, Kaftiobacteriaceae* and *Humicultoraceae*. These hydrogenases couple H_2_ oxidation to the reduction of NADP^+^ [40]. Seven *Thermofontiviventaceae* MAGs, 10 *Kaftiobacteriaceae* MAGs and 12 *Humicultoraceae* MAGs harbor NiFe 3c-type hydrogenases, suggesting they might use this complex to bifurcate electrons from H_2_ to ferredoxin and heterodisulphide [41]. Hydrogen is most likely the electron donor for MAGs encoding the complete WLP, as only reduced ferredoxin can provide electrons for the reduction of CO_2_ to CO. The oxygen-tolerant NiFe hydrogenase group 3d complex was identified in *Humicultoraceae* MAGs, suggesting they may maintain redox balance by interconverting electrons between NADH and H_2_ [40]. NiFe 1g-type hydrogenases were detected in *Sysuimicrobiaceae* and *Humicultoraceae* MAGs. These hydrogenases are suspected to liberate electrons for sulfur respiration [40]. Additionally, only MAGs within the *Humicultoraceae* encoded a NiFe 1f-type hydrogenase, which serves as an electron source to reduce reactive oxygen species [42]. In general, it is likely that these hydrogenases play a role in maintaining redox homeostasis and conserving energy.

Meanwhile, the gene encoding sulfide quinone oxidoreductase (*sqr*) was detected in 109 *Sysuimicrobiota* genomes. Sulfide quinone oxidoreductase plays a role in sulfide homeostasis and sulfide-dependent energy conservation [43], implying that *Sysuimicrobiota* can derive energy from sulfide oxidation. Furthermore, MAGs from all families, with the exception of *Segetimicrobiaceae*, contain genes for nitrite oxidoreductase (NxrAB)/nitrate reductase (NarGHI) complexes. Phylogenetic analysis showed that NxrA/NarG genes from *Kaftiobacteriaceae* and *Humicultoraceae* were closely related to known periplasmic nitrite oxidoreductase (NXR) of *Nitrospira, Nitrospina* and anammox bacteria (Fig. S19 and Supplementary Data), which suggests nitrite oxidation may be an alternative strategy for energy conservation [44,45].

### Acetogenesis in *Sysuimicrobiota*

Genes encoding acetyl-CoA synthetase (*acs*) were found in members of *Sysuimicrobiaceae, Thermofontiviventaceae, Kaftiobacteriaceae* and *Humicultoraceae*, implying the potential for acetogenesis. The wide distribution of *acs*, along with hydrogenases and enzymes involved in the WLP and RGP suggest members of the *Humicultoraceae* are acetogenic via the H_2_-dependent reduction of CO_2_ to acetate, while acetogenesis in *Sysuimicrobiaceae, Thermofontiviventaceae* and *Kaftiobacteriaceae* likely occurs via the RGP. The phosphate acetyltransferase (*pta*)-acetate kinase (*ack*) pathway is a distinct pathway to metabolize acetate, and is widely distributed in bacteria and in some archaea such as *Methanosarcina* [46] and *Bathyarchaeota* [47]. However, *pta* and *ack* were absent in all *Sysuimicrobiota* MAGs.

### Metagenomics-guided cultivation supports the proposed chemoautotrophic lifestyle in six genera of thermophilic *Sysuimicrobiota*

**Figure 3.**
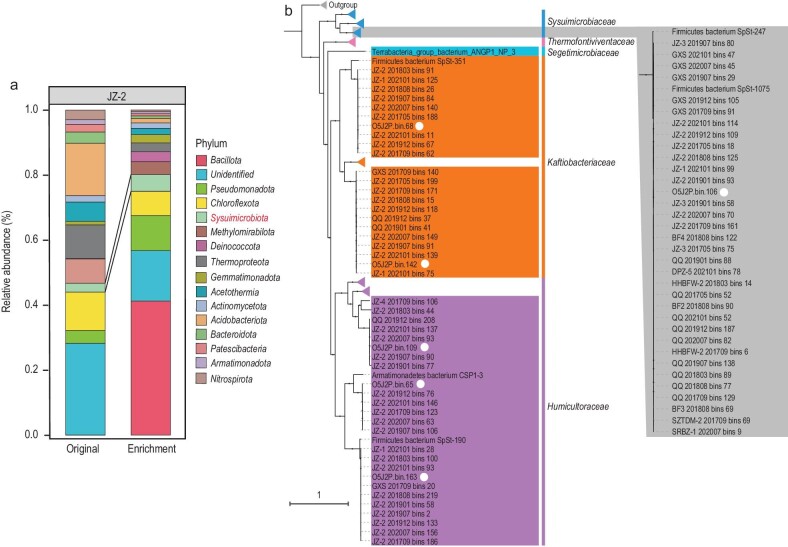
Laboratory enrichment of *Sysuimicrobiota*. (a) Microbial community composition of the original sample and chemoautotrophic enrichments based on 16S rRNA gene amplicons at the phylum level. (b) Phylogenetic placement of *Sysuimicrobiota* based on 31 concatenated bacterial marker genes. MAGs from the enrichment samples are shown with white circles. Bootstrap values were based on 1000 replicates and nodes with percentages >70% indicated as black circles.

To test our hypothesis that many *Sysuimicrobiota* are chemoautotrophs, we conducted chemoautotrophic enrichments at 45°C under anaerobic conditions, with sediments from JinZe hot spring (JZ-2; 25°26ʹ28″ N, 98°27ʹ36″ E), located in Tengchong, Yunnan Province, China, as the inoculum by using SYSUbac medium (Materials and Methods). The primary enrichment was transferred 10 times, after which FISH seems to have confirmed the presence of *Sysuimicrobiota* members in the enriched culture (Fig. S20). Subsequently, we monitored the microbial community composition using 16S rRNA gene amplicon sequencing (Fig. 3a) and genome-resolved metagenomics (Fig. 3b). Analysis of the 16S rRNA gene amplicon data revealed that *Bacillota, Pseudomonadota* and *Sysuimicrobiota* were enriched and became the top four abundant phyla alongside *Chloroflexota* in the enrichment culture, with the *Sysuimicrobiota* increasing from 2.73% to 5.91% (Fig. 3a). Based on GTDB-Tk and ANI results, six MAGs, which were recovered from a metagenome derived from the lab enrichment, belonged to six different genera within three different families of *Sysuimicrobiota* (Fig. 3b).

**Figure 4.**
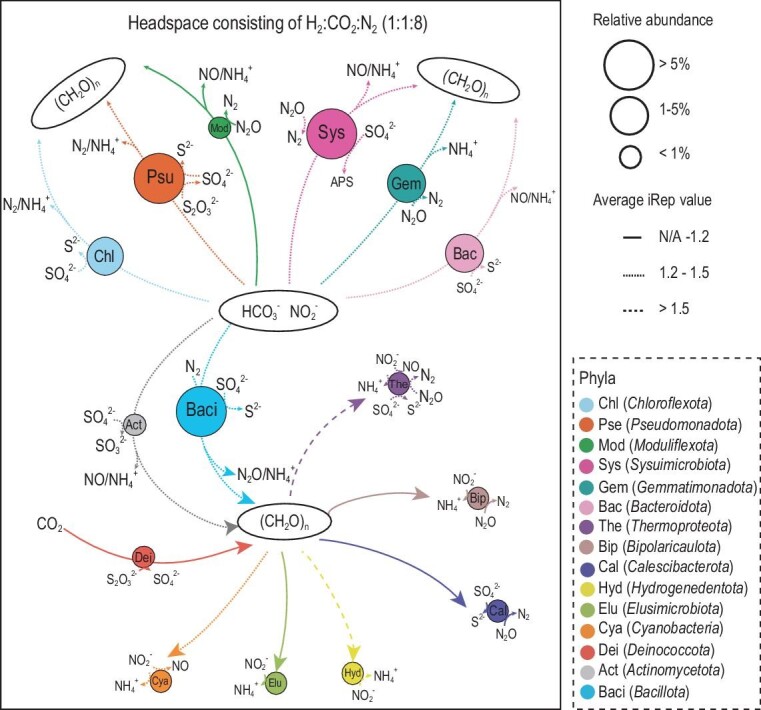
Genome-resolved metabolic models in the chemoautotrophic enrichment. The metabolic models are based on the 136 representative MAGs (Table S6). Phyla in the enrichment samples are shown with circles, and the size of circles indicate the relative abundance of phylum. The type of lines represent the average iRep values. Pse-*Pseudomonadota*, Sys-*Sysuimicrobiota*, Baci-*Bacillota*, Chl-*Chloroflexota*, Gem-*Gemmatimonadota*, Bac-*Bacteroidota*, Mod-*Moduliflexota*, Act-*Actinomycetota*, Dei-*Deinococcota*, The-*Thermoproteota*, Bip-*Bipolaricaulota*, Cal-*Calescibacterota*, Hyd-*Hydrogenedentota*, Elu-*Elusimicrobiota*, Cya-*Cyanobacteria*.

In the enrichment culture, *Sysuimicrobiota, Pseudomonadota* and *Bacillota* members were identified as the most abundant putative autotrophs (Fig. 4 and Table S6). The genes encoding the RGP were detected in all six enriched *Sysuimicrobiota* MAGs (herein proposed as *Tepidifontimicrobium thermophilum* O5J2P.bin.106, *Kaftiobacterium secundum* O5J2P.bin.68, *Calidihabitans tengchongensis* O5J2P.bin.142, *Humicultor tengchongensis* O5J2P.bin.65, *Geohabitans tengchongensis* O5J2P.bin.163 and *Fervidifonticultor tertius* O5J2P.bin.109), consistent with their predicted functions as autotrophs. Furthermore, the higher average iRep values (1.39) observed in the *Sysuimicrobiota* MAGs compared to other putative autotrophs indicate their fast growth rate under autotrophic conditions. In addition, genes encoding steps in the N and S cycles (*nxrA*/*narG, nirK, nosZ, sqr, sat, soxB* and *soxC*) (Fig. 4; Supplementary Data; Table S6) were detected in the enriched *Sysuimicrobiota* MAGs, suggesting multiple diverse roles in multiple biogeochemical cycles.

### Metatranscriptomic evidence of chemoautotrophy in *Sysuimicrobiota*

**Figure 5.**
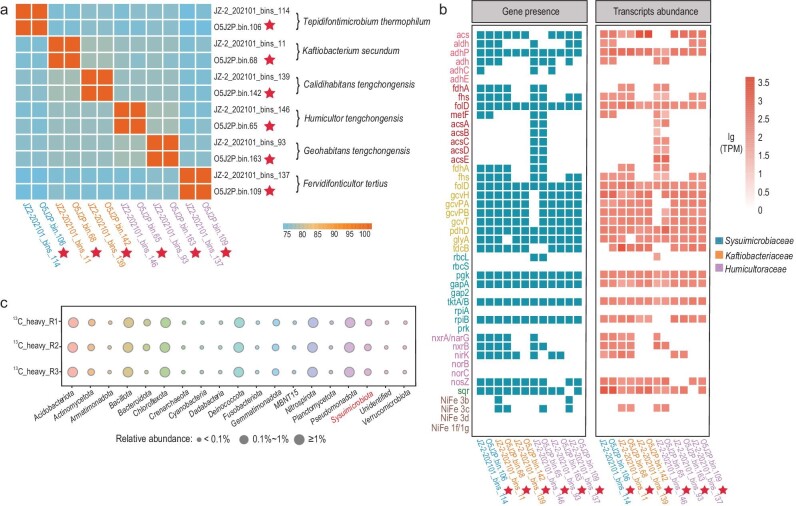
Gene expression and ^13^C-bicarbonate uptake by *Sysuimicrobiota*. (a) Average nucleotide identity (ANI) among MAGs from the enrichment samples and related members of *Sysuimicrobiota*. MAGs from the enrichment samples are shown with red stars. (b) The presence and expression of genes related to autotrophy, denitrification, sulfide oxidation and hydrogen metabolism in *Sysuimicrobiota*. (c) Community phylum composition in the heavy DNA fractions for three replicates following labeling with ^13^C-bicarbonate.

Genome-predicted functions of *Sysuimicrobiota*, including multiple autotrophic pathways and electron donors and acceptors, were further supported by metatranscriptomics data from the chemoautotrophic enrichment culture, together including 12 MAGs and 6 different species (Fig. 5a and b). Briefly, JZ-2_202101_bins_146 in the *Humicultoraceae* expressed the entire suite of genes for the WLP, and the WLP was partially expressed across all other 11 MAGs. Moreover, the GCS of the RGP transcripts were mapped to 10 of the 12 MAGs, and all genes encoding enzymes encoding the RGP were expressed in JZ-2_202101_bins_11. *Sqr*, encoding sulfide quinone oxidoreductase [43] was transcribed by 11 of the 12 MAGs, and *mvhA*, encoding an electron bifurcating NiFe 3c-type hydrogenase, was transcribed in MAGs affiliated with *Kaftiobacteriaceae* and *Humicultoraceae*. Additional details on key transcripts involved in carbon, nitrogen and sulfur metabolisms, oxidative phosphorylation, fermentation and response to environmental stresses are provided in Fig. 5b and Table S7.

In total, gene transcripts within the enrichment culture support the hypothesis that these *Sysuimicrobiota* are autotrophic via the WLP and RGP by using hydrogen and sulfide as electron donors.

### DNA-SIP identifies *Sysuimicrobiota* that incorporate ^13^C-bicarbonate

Given the enrichment of several *Sysuimicrobiota* under chemoautotrophic conditions and the expression of genes for the WLP and RGP, DNA-SIP was conducted to determine whether they can incorporate ^13^C from the ^13^C-NaHCO_3_ that was added to the enrichment culture. Following labeling and density-gradient centrifugation of the isolated DNA, quantitative polymerase chain reaction (qPCR) was used to determine the numbers of 16S rRNA genes across gradient density fractions, revealing slightly higher abundances of 16S rRNA genes in ‘heavy’ fractions (≥1.725 g mL^−1^) from incubations with ^13^C-DNA (Fig. S21). To reveal the identity of the taxa that incorporated the ^13^C atoms, 16S rRNA gene amplicon sequencing was performed using DNA from one of the heavy fractions (1.732 g mL^−1^) (Table S8). Five *Sysuimicrobiota* ASVs (ASV1, ASV2, ASV3, ASV4 and ASV5) were detected in the heavy DNA fraction with a relative abundance of 0.61%, 0.41% and 0.65% (Fig. 5c; Table S9). Phylogenetic analyses of 16S rRNA genes from *Sysuimicrobiota* MAGs and the *Sysuimicrobiota* amplicon sequence variants (ASV) showed that the labelled *Sysuimicrobiota* ASVs were affiliated with three species belonging to two families, *Humicultoraceae* and *Sysuimicrobiaceae* (Fig. S22). Although only one ‘heavy’ fraction was used for 16S rRNA gene amplicon sequencing, the *Sysuimicrobiota* was notable because it was highly enriched in the chemoautotrophic enrichment versus the original sample (5.91% vs. 2.73%), and genes for chemoautotrophic traits were highly expressed.

Although the chemoautotrophic lifestyle of *Sysuimicrobiota* remains unclear due to the absence of pure culture, the metagenomic data, along with the enrichment of several different *Sysuimicrobiota* under chemoautotrophic conditions that express autotrophic pathways and incorporate ^13^C atoms from ^13^C-NaHCO_3_, provide evidence that *Sysuimicrobiota* have chemoautotrophic potential and suggest they may play important roles in various biogeochemical cycles.

### Evolutionary history of *Sysuimicrobiota*

**Figure 6.**
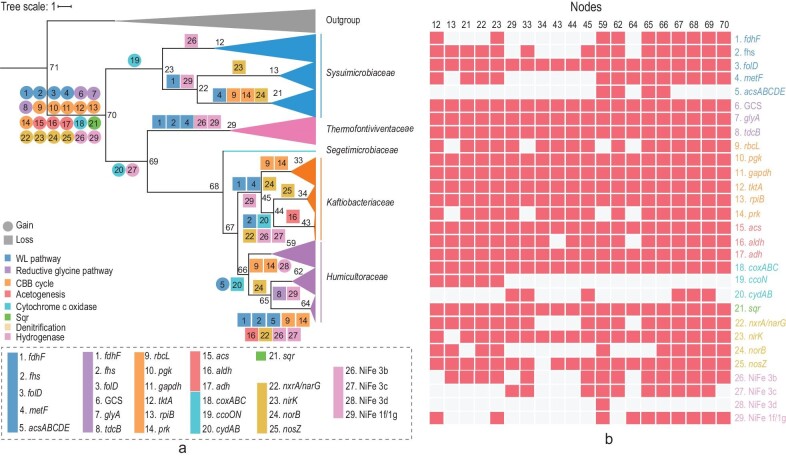
Reconstruction of *Sysuimicrobiota* with regard to evolutionary history. (a) Inferred gene gain and loss events related to carbon fixation and nitrogen metabolism in *Sysuimicrobiota*. The Bayesian tree topology was determined by MrBayes [74]. (b) The presence and absence of genes in *Sysuimicrobiota* MAGs. Numbers above the columns are nodes corresponding to those on the left side (a).

To infer the evolutionary history of *Sysuimicrobiota*, the Dollo parsimony method in COUNT was implemented to identify the gene gain and loss events by mapping gene orthologous groups to a Bayesian tree of 31 concatenated bacterial marker genes (Fig. 6) that was consistent with the maximum-likelihood trees presented earlier (Fig. 1a and Fig. S2). Genes encoding the low-affinity terminal oxidase (*coxABC*), *acs, aldh* and *adh* were widespread in *Sysuimicrobiota* and were predicted to be present in the ancestor of the phylum, indicating a facultatively anaerobic lifestyle for the ancestor. The lack of genes for the CODH/ACS complex in *Sysuimicrobiota* and their absence in the ancestor of the phylum is consistent with a recent gene transfer event into members of the *Humicultoraceae* that was suggested by the CODH/ACS phylogeny (Figs S7–S11). Similarly, RuBisCO genes were not ancestral in the phylum and arose from two HGT events. In contrast, the RGP is present in all families and is predicted to have been present in the common ancestor of *Sysuimicrobiota*, consistent with the monophyly of *Sysuimicrobiota* GcvPA, GcvPB, GcvT, GcvH and PdhD in phylogenetic trees (Figs S12–S16).

Nitrite reductase (*nirK*) and nitrous oxide reductase (*nosZ*) were mostly acquired horizontally in *Sysuimicrobiaceae, Thermofontiviventaceae, Kaftiobacteriaceae* and *Humicultoraceae* (Figs S23 and S24). Additionally, phylogenetic analysis based on NxrA/NarG showed that enzymes from *Sysuimicrobiaceae* and *Thermofontiviventaceae* arose from two HGT events (Fig. S19). The *norB* genes were mostly acquired horizontally in *Sysuimicrobiaceae* MAGs. Thus, denitrification is exclusively detected in *Sysuimicrobiaceae*. Similarly, *sqr* (Fig. S25) and hydrogenase (Fig. S18) genes were not ancestral in the phylum and arose from HGT events.

The common ancestor of extant *Sysuimicrobiota* was likely facultatively anaerobic and capable of acetogenesis and autotrophy via the RGP. Genes related to the CBB, WLP and denitrification were obtained by HGT events, leading to a wide range of functions within this phylum.

## DISCUSSION

Members of CSP1-3 are lacking cultured representatives and their phylogeny has remained imprecise. In this study, we incorporated a large number of CSP1-3 MAGs including published (15 MAGs) and newly obtained (97 MAGs) ones from a hot spring, which showed the CSP1-3 nested in the phylogenomic tree as a single phylum along with the declaration of 1 class, 1 order, 5 families, 11 genera and 25 species, rather than the member of *Armatomonadota* [9], and herein designated as *Sysuimicrobiota* phy. nov. Furthermore, higher relative abundance of *Sysuimicrobiota* was observed in hot springs. Thus, our findings considerably expand the *Sysuimicrobiota* species tree and support that hot springs are a rich source of novel microbial branches on the tree of life [33,48–51].

Genome-centric metagenomics provide insights into the functional potential of *Sysuimicrobiota.* The presence of *acs, aldh* and *adh* among MAGs from *Sysuimicrobiota* suggests that *Sysuimicrobiota* are likely able to ferment and can survive under anaerobic conditions. This finding may explain the increase in abundance of *Sysuimicrobiota* with soil depth [52–54]. Additionally, the presence of a complete electron transport chain for aerobic respiration reflects an aerobic lifestyle, a discovery made for the first time in our study. Members of *Sysuimicrobiota* contain complete gene sets for WLP, which is in line with the previous studies [9,20]. However, WLP is limited to members of *Humicultoraceae*, indicating that the presence of complete WLP is not prevalent in the phylum *Sysuimicrobiota*. The autotrophy potential of *Sysuimicrobiota* is also indicated by the presence of the RGP and CBB pathway, expanding our understanding of *Sysuimicrobiota.* Furthermore, members of *Sysuimicrobiota* play important roles in biogeochemical cycles of nitrogen and sulfur, which are also supported by previous studies [9,19]. Considering all these findings, members of *Sysuimicrobiota* exhibit a versatile lifestyle, which is mainly attributed to the ability to adapt to various habitats [9–17,19] and the global distribution of *Sysuimicrobiota*.

In the current study, through genome-resolved metagenomics, we constructed a robust phylogeny and taxonomy for the phylum, which enabled a thorough investigation of the metabolic potential and evolutionary history of this phylum. Our study is the first comprehensive view into the diversity of *Sysuimicrobiota*, and delivers a blueprint of its functions, ecology and evolution. An important prediction arising from the metagenomic analysis is that a significant proportion of *Sysuimicrobiota* members are facultatively anaerobic chemoautotrophs, with diverse functions in nitrogen and sulfur cycles. This prediction was then tested by enriching *Sysuimicrobiota* under autotrophic conditions. These enriched *Sysuimicrobiota* belong to six different species and represent all three *Sysuimicrobiota* families. By mapping transcripts to the WLP and RGP and tracing ^13^C-bicarbonate into their DNA, we provide evidence of autotrophy potential. We encourage future cultivation attempts and additional molecular ecology studies based on the data and interpretations presented here in order to better understand the physiology and ecology of these organisms.

## PROTOLOGUES

### Description of *Sysuimicrobiota* phy. nov.

*Sysuimicrobiota* (Sy.su.i.mic.ro'bi.o'ta. N.L. neut. n. *Sysuimicrobium* type genus of the phylum; L. suff. –*ota* ending to denote a phylum; N.L. pl. neut. n. *Sysuimicrobiota* the *Sysuimicrobium* phylum).

Type genus: *Sysuimicrobium*.

### Description of *Sysuimicrobiia* classis nov.

*Sysuimicrobiia* (Sy.su.i.mic.ro'bi.ia. N.L. neut. n. *Sysuimicrobium* referring to the type genus of the class; L. suff. –*ia* ending to denote a class; N.L. neut. pl. n. *Sysuimicrobiia* the *Sysuimicrobium* class).

The description of the class is the same as for the order *Sysuimicrobiales*.

Type order: *Sysuimicrobiales*.

### Description of *Sysuimicrobiales* ord. nov.

*Sysuimicrobiales* (Sy.su.i.mi.cro'bi.a'les. N.L. neut. n. *Sysuimicrobium* type genus of the order; L. suff. –*ales* ending to denote an order; N.L. fem. pl. n. *Sysuimicrobiales* the *Sysuimicrobium* order).

The order *Sysuimicrobiales* comprised of five families *Sysuimicrobiaceae* fam. nov., *Thermofontivivantaceae* fam. nov., *Segetimicrobiaceae* fam. nov., *Kaftiobacteriaceae* fam. nov. and *Humicultoraceae* fam. nov.

Type family: *Sysuimicrobiaceae*.

### Description of *Sysuimicrobiaceae* fam. nov.

*Sysuimicrobiaceae* (Sy.su.i.mi.cro.bi.a.ce'ae. N.L. neut. n. *Sysuimicrobium* type genus of the family; L. suff. –*aceae* ending to denote a family; N.L. fem. pl. n. *Sysuimicrobiaceae* the *Sysuimicrobium* family).

The family at present contains three genera *Sysuimicrobium* gen. nov., *Caldifonticola* gen. nov. and *Tepidifontimicrobium* gen. nov.

Type genus: *Sysuimicrobium*.

### Description of *Sysuimicrobium* gen. nov.

*Sysuimicrobium* (Sy.su.i.mi.cro.bi.um. N.L. neut. n. *microbium* microbe; N.L. neut. n. *Sysuimicrobium*, arbitrary name from the acronym of Sun Yat-Sen University (SYSU) where the identification of the microbial metagenome took place).

Type species: *Sysuimicrobium tengchongense*.

### Description of *Sysuimicrobium tengchongense* sp. nov.

*Sysuimicrobium tengchongense* (teng.cong.en'se. N.L. neut. adj. *tengchongense* referring to Tengchong).

Type material: BF2_201808_bins_94, obtained from the metagenome assembly of a hot spring sample from Tengchong, P.R. China.

## MATERIALS AND METHODS

### Sample collection and metagenomic analysis

A total of 39 surface sediment samples (top 1 cm) were collected by using sterile spoons from hot springs located in Tengchong County, Yunnan Province, China. The distance between sampling points ranges from 2 m to 59.13 km (Table S1). At the time of sampling, these hot springs spanned a wide range of physicochemical parameters with temperatures ranging from 36 to 91.3°C and pH ranging from 6.1 to 8.7. Table S1 includes sampling dates, GPS coordinates, temperature, pH and other geochemical properties. Physicochemical analyses were conducted as detailed in the ‘Physicochemical analysis’ subsection of Supplementary Methods. For DNA and RNA extraction, samples were stored on dry ice in the field and shipped to the laboratory for storage at -80°C. Total community DNA from the samples was extracted using the FastDNA Spin kit (MP Biomedicals, California, USA) according to the manufacturer's protocols. DNA quantity and quality were checked using a NanoDrop® ND-2000 spectrophotometer (Thermo Fisher Scientific, Waltham, Massachusetts, USA) and 1.0% agarose gel electrophoresis. A next-generation sequencing library with an insert size of 350 bp was constructed (Supplementary Data), and metagenomes were sequenced by using the Illumina HiSeq X Ten platform (2 × 150 bp) at Azenta, Suzhou, China.

Quality control for raw reads generated by Illumina HiSeq were conducted as previously described [55] using custom Perl scripts available at https://github.com/hzhengsh/qualityControl. Briefly, adapter-contaminated and duplicated reads were first eliminated. Low-quality reads with a significant excess of ‘N’ (≥5% of the read) were removed. The remaining reads with a quality score of <20 were trimmed at both ends. After that, the high-quality reads from each sample were assembled using SPAdes (version 3.9.0) [56] with the parameters -meta -k 21, 33, 55, 77, 99, 127 and then mapped to the assembled scaffolds using BBMap (version 38.85; http://sourceforge.net/projects/bbmap/) with the parameters ‘k = 15 minid = 0.9 build = 1’. Binning of the 39 metagenomes was conducted individually using only scaffolds with length > 2.5 kbp using MetaBAT2 [57]. The completeness, contamination and heterogeneity of the retrieved bins from MetaBAT2 were determined based on lineage-specific conserved marker gene sets in each bin by using CheckM [58] (v2.12.1). Prodigal [59] (v2.6.3) with the ‘-p single’ parameter was used to predict putative protein-coding sequences (CDSs). Functional annotation and metabolic reconstructions were performed by querying the predicted CDS against the databases, including NCBI-nr, KEGG and eggnog, using DIAMOND [60] (v0.7.9) and by applying E-values < 1e-10. The predicted CDSs were uploaded to the KEGG automatic annotation server [61] with ‘for prokaryotes’ and ‘bidirectional best hit’ options for pathway analysis. Genomes were processed using tRNAscan-SE version 2.0.2 [62] and RNAmmer version 1.2 [63] for RNA gene prediction.

### Phylogenetic analysis

In total, 112 *Sysuimicrobiota* MAGs including 97 from existing hot spring sediment metagenomes (Table S1) and 15 from currently publicly available databases (NCBI) with >80% genomic completeness and <5% genomic contamination were collected for the phylogenomic tree reconstruction by using three different marker protein sets (https://github.com/liulan9/Sysuimicrobiota). GTDB-Tk v2 [64] was used for the preliminary construction of the phylogenetic tree. In detail, a multiple sequence alignment of 120 bacteria-specific conserved marker proteins were generated by GTDB-Tk v2, and then the phylogenetic analysis of these 112 genomes plus representatives of each GTDB phylum was performed by FastTree [65]. To further resolve the phylogenetic affiliations of the MAGs within the CSP1-3, five type strains from the *Armatimonadota* located on the periphery of CSP1-3 based on the genome phylogeny determined by GTDB-Tk v2, were chosen as the outgroup. Sixteen ribosomal protein sequences (L2, L3, L4, L5, L6, L14, L15, L16, L18, L22, L24, S3, S8, S10, S17 and S19) [6] were identified and aligned using MUSCLE v3.8.31 by iterating 100 times [66]. Poorly aligned regions were eliminated using TrimAl.v1.4 with the parameters ‘-gt 0.95 -cons 50’ [67]. Then, multiple alignments were concatenated using a Perl script (https://github.com/nylander/catfasta2phyml), and the phylogenetic tree was generated using IQ-Tree [68] (v1.6.10). Furthermore, a total of 31 conserved marker proteins (DnaG, Frr, InfC, NusA, Pgk, PyrG, RplA, RplB, RplC, RplD, RplE, RplF, RplK, RplL, RplM, RplN, RplP, RplS, RplT, RpmA, RpoB, RpsB, RpsC, RpsE, RpsI, RpsJ, RpsK, RpsM, RpsS, SmpB and Tsf) were selected and identified by AMPHORA2 [21], and a phylogenetic tree was constructed as described previously [69]. A phylogenetic tree of 16S rRNA gene sequences was carried out by integrating the 16S rRNA gene sequences retrieved from the genomes of CSP1-3 into the pre-aligned SILVA tree (‘LTPs132_SSU’ [70]) with maximum parsimony in ARB (from Latin *arbor*, tree) [71]. Phylogenetic analyses of proteins of interest, such as CODH/ACS, RbcL (ribulose-1,5-bisphosphate carboxylase/oxygenase large subunit) and hydrogenases, were performed as described by Jiao *et al.* [33]. Phylogenetic trees of NarG/NxrA, GcvPA, GcvPB, GcvT, GcvH, PdhD, NirK, NosZ and Sqr were constructed as mentioned above using the best model. Sequences for the phylogenetic analyses of proteins are provided in the supplementary tables (Tables S10–S30). Finally, all phylogenetic trees were visualized using the interactive Tree Of Life (iTOL) tool v6 [72].

### Biogeographic distribution of *Sysuimicrobiota*

To better understand the environmental distribution of *Sysuimicrobiota*, 16S rRNA gene sequences from members of the *Sysuimicrobiota* were used as queries in the IMNGS database [25]. The similarity threshold was set at 97% and the sequence length threshold was set at 200 bp, which are suggested by IMNGS [25] for species-level investigation, because most of the sequences in the IMNGS database are short reads rather than full-length 16S rRNA gene sequences. All the samples from different niches (e.g. soil, biofilm, water and hot spring) in the IMNGS server were assigned to study the abundant and biogeographic characterizations of *Sysuimicrobiota*, and only hits with >0.1% relative abundance were considered. To further confirm the 16S rRNA genes identified as *Sysuimicrobiota* by IMNGS, we blast the 16S rRNA genes from our *Sysuimicrobiota* MAGs against the SILVA database. The results showed that almost all of the 16S rRNA genes from the MAGs were aligned as members of the GAL15 group, with identity values of best-hits ranging from 80.26% to 85.71%. The exceptions, all belonging to the *Thermofontiviventaceae*, were not identified at the phylum level. No sequences were assigned to the phylum *Armantimonadota*.

### Comparative genomics

For comparative genomics, genomes with 80% completeness were taken into account, including 112 *Sysuimicrobiota* MAGs. Meanwhile, five type strains from the *Armatimonadota* were chosen as the outgroup. ANI among the *Sysuimicrobiota* genomes was calculated by using the pyANI with the blast method [73]. AAI was calculated as a mean similarity of orthologous genes, as earlier indicated [69]. To address the evolutionary history of *Sysuimicrobiota*, a Bayesian tree based on the 31 marker protein alignment was constructed by MrBayes [74] with parameters (ngen = 3 000 000, Nruns = 2, Nchains = 4, diagnfreq = 1000, relburnin = yes, burninfrac = 0.25, samplefreq = 100, printfreq = 100), and evolutionary histories were inferred using COUNT v9.1106 with Dollo parsimony [75].

### Metagenomics-guided laboratory cultivation

Based on the results of our analysis of MAGs and physicochemical factors where *Sysuibacterota* are abundant, we selected sample locations and designed strategies to cultivate *Sysuimicrobiota* [5,76, 77]. Sediments from JinZe-2 (JZ-2) hot spring (98.46000E, 25.44111N) were selected as the inoculum due to the stable presence of *Sysuimicrobiota* and its lower temperature (∼45°C). The top 1 cm of sediment from JZ-2 hot spring was placed into a single sterile Balch tube using a sterile spoon and transported to the laboratory without redox or temperature control. Anaerobic enrichments were set up under a dark condition at 45°C and seeded with 200 g of hot spring sediment. The chemoautotrophic enrichment medium (SYSUbac medium) contained the following components (per liter): 330 mg (NH_4_)_2_SO_4_; 27.2 mg KH_2_PO_4_; 300 mg MgSO_4_·7H_2_O; 180 mg CaCl_2_·2H_2_O. The pH of the media was adjusted to 7.0. After autoclaving, trace 1 (5 g EDTA; 5 g FeSO_4_; 1 L ddH_2_O), trace 2 (15 g EDTA; 0.43 g ZnSO_4_·7H_2_O; 0.24 g CoCl_2_·6H_2_O; 0.99 g MnCl_2_·4H_2_O; 0.25 g CuSO_4_·5H_2_O; 0.22 g NaMoO_4_·2H_2_O; 0.19 g NiCl_2_·6H_2_O; 0.21 g NaSeO_4_·10H_2_O; 0.014 g H_3_BO_4_;1 L ddH_2_O), KHCO_3_, Na_2_S and NaNO_2_ were added from sterile stock solutions to obtain final concentrations of 1 mL/L, 1 mL/L, 500 mg/L, 500 mg/L and 345 mg/L, respectively. The headspace consisted of N_2_: H_2_: CO_2_ (80 : 10 : 10, v/v/v), which was flushed and replenished every month.

### 16S rRNA gene amplicon, metagenomic and metatranscriptomic analyses of enrichment sample

After 150 days of enrichment, cells were collected by centrifugation (12 000 g, 15 min). The extraction of genomic DNA, metagenomic sequencing and analyses were performed as described above. To investigate the replication rate *in situ* of each MAG, the index of replication (iRep) v1.10 was conducted using thresholds (min cov. = 5, min wins. = 0.98, min r^2 = 0.9, max fragments/Mbp = 175, GC correction min r^2 = 0.0) as described [33]. The bacterial V4 region of the 16S rRNA gene was amplified by 515F (5′-GTGCCAGCMGCCGCGGTAA-3′) and 806R (5′- GGACTACHVGGGTWTCTAAT-3′) [78] (Supplementary Methods) and sequenced on the Illumina MiSeq platform of Azenta, Suzhou, China. Data analysis of the 16S rRNA gene sequences generated by MiSeq was performed using quantitative insights into microbial ecology 2 (QIIME2, v2019.1) [79] (https://github.com/liulan9/Sysuimicrobiota). Briefly, DADA2 [80] was used to filter, trim, denoise and merge sequences. Chimeric sequences were identified and removed. Taxonomy classifications of bacterial 16S rRNA genes at the phylum level were assigned to chimeric-free sequences using q2-feature-classifier [81] classify-sklearn Naïve Bayes taxonomy classifier against the SILVA v138 99% operational taxonomic units (OTUs) reference sequences.

Total cellular RNA was extracted using the RNeasy Mini kit (Qiagen, Dusseldorf, Germany) according to the manufacturer's instructions. Total RNA was transported to Azenta, Suzhou, China on dry ice for subsequent rRNA subtraction, cDNA synthesis, library construction and sequencing with an Illumina HiSeq. Raw metatranscriptomic reads were pre-processed in the same manner as metagenomic reads. The rRNA sequences were removed by SortMeRNA (version 2.180) [82]. Subsequently, these high-quality transcriptomic reads were mapped to the predicted protein-coding genes from the metagenomic co-assembly using BBMap, and the expression level for each gene was normalized to transcripts per million mapped reads (TPM).

### FISH detection and stable isotope probing incubation

FISH was performed as described previously to detect the *Sysuimicrobiota* in the enrichment culture [83] (Supplementary Methods). The FISH probe (5'-TGTTGGGGGGTTACTCCC-3') for *Sysuimicrobiota* was designed using ARB software [71], and verified by SILVA (a coverage of 67.4% for the GAL15 with 0 mismatches). DNA-SIP was conducted using ^13^C-labeled NaHCO_3_ to determine whether the *Sysuimicrobiota* species were autotrophic. Briefly, 60 mL of enrichment culture was mixed with 240 mL broth as previously described in a serum bottle (500 mL) in an anaerobic chamber. The headspace was purged with N_2_ : H_2_ : CO_2_ (80 : 10 : 10, v/v/v), and then the bottle was sealed immediately with butyl rubber septa and aluminum caps. All cultures were incubated at 45°C without agitation for 25 days. Total genomic DNA from the samples was extracted using the FastDNA Spin kit (MP Biomedicals, California, USA). DNA quantity and quality were checked using a NanoDrop® ND-2000 spectrophotometer (Thermo Fisher Scientific, Waltham, Massachusetts, MA). The DNA from SIP incubation was separated into ‘light’ (^12^C_light) and ‘heavy’ (^13^C_heavy) fractions using CsCl gradient centrifugation (Supplementary Methods) and then analysis of community DNA was performed as previously described [84] by quantifying the 16S rRNA gene copy numbers (Supplementary Methods). Finally, the 16S rRNA genes of DNA from ^13^C_heavy was amplified with primer pair 515F/806R. Subsequently, amplicon sequencing and analysis of raw Illumina fastq files were performed as described above.

## Supplementary Material

nwae378_Supplemental_Files

## Data Availability

The MAGs from the enrichment and existing hot spring metagenome data sets in this paper have been deposited under NCBI PRJNA973840. Raw reads for metagenomic and metatranscriptomic data sets are available under this project with the SRA accessions SRR27730012-SRR27730050 and SRR27732999, respectively. Additionally, all the amplicon sequences were uploaded to the NCBI database under the same project. Scripts have been uploaded at https://github.com/liulan9/Sysuimicrobiota and https://github.com/hzhengsh/qualityControl.
