# Supplementary material for: Insights into chemoautotrophic traits of a prevalent bacterial phylum CSP1-3, herein Sysuimicrobiota
Source: Natl Sci Rev. 2024 Oct 23;11(11):nwae378. doi: 10.1093/nsr/nwae378 (PMC11604079; doi:10.1093/nsr/nwae378)
Supplement: nwae378_Supplemental_Files [file nwae378_supplemental_files.zip › Supplementary_Information.docx]

**SUPPLEMENTARY INFORMATION**

**Insights into chemoautotrophic traits of a prevalent bacterial phylum CSP1-3, herein *Sysuimicrobiota***

**Lan Liu^1^, Zheng-Han Lian^1^, Ai-Ping Lv^1^, Nimaichand Salam^2^, Jian-Chao Zhang^3^, Meng-Meng Li^1^, Wei-Min Sun^4^, Sha Tan^1^, Zhen-Hao Luo^1^, Lei Gao^5^, Yang Yuan^1^, Yu-Zhen Ming^1^, Yu-Ting OuYang^1^, Yu-Xian Li^6^, Ze-Tao Liu^1^, Chao-Jian Hu^1^, Ying Chen^1^, Zheng-Shuang Hua^6^, Wen-Sheng Shu^7,8^, Brian P. Hedlund^9,10^, Wen-Jun Li^1,5^*, Jian-Yu Jiao^1^***

^1^State Key Laboratory of Biocontrol, Guangdong Provincial Key Laboratory of Plant Stress Biology and Southern Marine Science and Engineering Guangdong Laboratory (Zhuhai), School of Life Sciences, Sun Yat-Sen University, Guangzhou, 510275, China

^2^National Agri-Food Biotechnology Institute, Sector-81 (Knowledge City), Mohali, 140306, Punjab, India

^3^School of Earth System Science, Institute of Surface-Earth System Science, Tianjin University, Tianjin, 30072, China

^4^National-Regional Joint Engineering Research Center for Soil Pollution Control and Remediation in South China, Guangdong Key Laboratory of Integrated Agro-environmental Pollution Control and Management, Institute of Eco-environmental and Soil Sciences, Guangdong Academy of Sciences, Guangzhou 510650, China

^5^State Key Laboratory of Desert and Oasis Ecology, Key Laboratory of Ecological Safety and Sustainable Development in Arid Lands, Xinjiang Institute of Ecology and Geography, Chinese Academy of Sciences, Urumqi, 830011, China

^6^Chinese Academy of Sciences Key Laboratory of Urban Pollutant Conversion, Department of Environmental Science and Engineering, University of Science and Technology of China, Hefei 230026, PR China

^7^Institute of Ecological Science, Guangzhou Key Laboratory of Subtropical Biodiversity and Biomonitoring, Guangdong Provincial Key Laboratory of Biotechnology for Plant Development, School of Life Sciences, South China Normal University, Guangzhou, 510631, China

^8^Guangdong Provincial Key Laboratory of Chemical Pollution, South China Normal University, Guangzhou, 510006, China

^9^School of Life Sciences, University of Nevada Las Vegas, Las Vegas, NV 89154, USA

^10^Nevada Institute of Personalized Medicine, University of Nevada Las Vegas, Las Vegas, NV 89154, USA

***Correspondence:**

Jian-Yu Jiao, Email: jiaojy5@mail.sysu.edu.cn

Wen-Jun Li, Email: liwenjun3@mail.sysu.edu.cn

TABLE OF CONTENTS

[SUPPLEMENTARY TEXT 5](#_Toc164454935)

[*SUPPLEMENTARY METHODS* *5*](#_Toc164454936)

[**Physicochemical analysis** **5**](#_Toc164454937)

[**Metagenomic library construction** **5**](#_Toc164454938)

[**PCR amplification of 16S rRNA gene** **6**](#_Toc164454939)

[**Metatranscriptomic sequencing** **6**](#_Toc164454940)

[**Fluorescence *in situ* hybridization (FISH)** **6**](#_Toc164454941)

[**DNA-SIP gradient fractionation** **7**](#_Toc164454942)

[**Quantitative PCR of 16S rRNA genes** **7**](#_Toc164454943)

[*SUPPLEMENTARY RESULTS* *8*](#_Toc164454944)

[**Nitrogen cycle in *Sysuimicrobiota*** **8**](#_Toc164454945)

[**Transcriptomic profile of nitrogen cycle** **8**](#_Toc164454946)

[**The reconstruction of nitrogen and sulfur metabolism in the enriched treatment** **9**](#_Toc164454947)

[*NOMENCLATURE OF MEMBERSIN SYSUIMICROBIOTA* *10*](#_Toc164454948)

[SUPPLEMENTARY FIGURES 19](#_Toc164454949)

[Supplementary Fig. S1. Phylogenetic tree showing the relationships of *Sysuimicrobiota* and other phyla. 19](#_Toc164454950)

[Supplementary Fig. S2. The phylogenetic tree of 16S rRNA gene sequences. 20](#_Toc164454950)

[Supplementary Fig. S3. Phylogenetic placements of *Sysuimicrobiota* MAGs based on 16 ribosomal proteins. 21](#_Toc164454951)

[Supplementary Fig. S4. Average amino acid identity (AAI) shared among genomes of *Sysuimicrobiota*. 22](#_Toc164454952)

[Supplementary Fig. S5. Average amino nucleotide identity (ANI) shared among genomes of *Sysuimicrobiota*. 23](#_Toc164454953)

[Supplementary Fig. S6. The core metabolic pathways with the presence/absence of genes of the five novel families. 24](#_Toc164454954)

[Supplementary Fig. S7. Maximum-likelihood phylogeny of concatenated AcsDABCE. 25](#_Toc164454955)

[Supplementary Fig. S8. Maximum-likelihood phylogeny of concatenated AcsAB. 26](#_Toc164454956)

[Supplementary Fig. S9. Maximum-likelihood phylogeny of concatenated AcsABC. 27](#_Toc164454957)

[Supplementary Fig. S10. Maximum likelihood phylogeny of concatenated AcsDEC. 28](#_Toc164454958)

[Supplementary Fig. S11. Maximum likelihood phylogeny of concatenated AcsDABC. 29](#_Toc164454959)

[Supplementary Fig. S12. Phylogenetic tree of GcvPA protein sequences. 30](#_Toc164454960)

[Supplementary Fig. S13. Phylogenetic tree of GcvPB protein sequences. 31](#_Toc164454961)

[Supplementary Fig. S14. Phylogenetic tree of GcvT protein sequences. 32](#_Toc164454962)

[Supplementary Fig. S15. Phylogenetic tree of GcvH protein sequences. 33](#_Toc164454963)

[Supplementary Fig. S16. Phylogenetic tree of PdhD protein sequences. 34](#_Toc164454964)

[Supplementary Fig. S17. The phylogenetic tree of RuBisCO large subunit. 35](#_Toc164454965)

[Supplementary Fig. S18. Phylogenetic tree of groups 1, 2 and 3 [NiFe] hydrogenases catalytic subunits. 36](#_Toc164454966)

[Supplementary Fig. S19. The phylogenetic tree of NxrA/NarG. 37](#_Toc164454967)

[Supplementary Fig. S20. Fluorescence in situ hybridization (FISH) images of *Sysuimicrobiota* in enrichment culture. 38](#_Toc164454968)

[Supplementary Fig. S21. Quantification of total bacterial 16S rRNA genes across density fractions. 39](#_Toc164454969)

[Supplementary Fig. S22. The phylogenetic tree based on 16S rRNA gene sequences of *Sysuimicrobiota* ASVs in the heavy DNA fractions of the ^13^C treatment and *Sysuimicrobiota* MAGs in this study. 40](#_Toc164454970)

[Supplementary Fig. S23. The phylogenetic tree of NirK. 41](#_Toc164454971)

[Supplementary Fig. S24. The phylogenetic tree of NosZ. 42](#_Toc164454972)

[Supplementary Fig. S25. The phylogenetic tree of Sqr. 43](#_Toc164454973)

# **SUPPLEMENTARY TEXT**

## **SUPPLEMENTARY METHODS**

### **Physicochemical analysis**

The geographical coordinates are interpreted by GPS devices. Temperature and pH were measured *in situ* using a portable multi-meter (HACH58258; HACH, Loveland, Colorado, USA). NH_4_^+^, NO_2_^-^ and NO_3_^-^ were measured according to the methods as described previously [1]. Briefly, sediments were dried, homogenized and filtered through a 2.0 mm sieve. Then, NH_4_^+^, NO_2_^-^ and NO_3_^-^ were extracted from 1 g of sieved sediments by shaking for 1 h at room temperature with 10 mL of 2 mol/L KCl, and finally measured using an ion chromatograph meter (ICS-600; Thermo Fisher Scientific, Waltham, Massachusetts, USA). Total sedimentary phosphorus (TP) was measured colorimetrically by the ascorbic acid-molybdate blue method after 2 h of combustion (500°C) and 16 h of extraction with 1M HCl [2]. The total carbon (TC) and total nitrogen (TN) of sediment samples were dried at 70 ºC until reaching a constant weight and finely ground, then measured by an elemental analyzer (TOC-V CPN; SHIMADZU, Tokyo, Japan). The concentration of sulfate (SO_4_^2-^) was determined using a BaSO_4_-based turbidimetric method [3]. Heavy metals (including total Cu, Zn, Pb, Fe, Cr) were measured using inductively coupled plasma optical emission spectrometry (ICP-OES; Optima 46 2100DV; Perkin-Elmer, Waltham, Massachusetts, USA).

### **Metagenomic library construction**

200 μg genomic DNA was randomly fragmented, and the fragments were treated with End Prep Enzyme Mix (Vazyme, Nanjing, China) for end repairing, 5’ phosphorylation and 3’ adenylated. Size selection of adaptor-ligated DNA was then performed by DNA Cleanup beads (Vazyme, Nanjing, China). Each sample was then amplified by PCR for 8 cycles using P5 and P7 primers to enrich the adapter-ligated DNA fragments. The PCR products were cleaned up and validated using an Agilent 2100 Bioanalyzer (Agilent, Santa Clara, California, USA). Finally, the libraries with an insert size of 350 bp were constructed, and metagenome data was sequenced by Illumina HiSeq X Ten platform (2 × 150 bp) at Azenta, Suzhou, China.

### **PCR amplification of 16S rRNA gene**

The bacterial V4 region of the 16S rRNA gene was amplified by primer pair 515F (5′-GTGCCAGCMGCCGCGGTAA-3′)/806R (5′-GGACTACHVGGGTWTCTAAT-3′) [4]. PCR reaction conditions were as follows: 94 ºC for 3 min; 16 cycles of 94 ºC for 10 s, 57 ºC for 90 s followed by 72 ºC for 15 s; and a final 5 min extension at 72 ºC. Blank controls containing no template DNA were performed under the same PCR procedure, and no PCR product was visible on agarose gels for negative controls. High-throughput paired-end Illumina MiSeq sequencing (2 × 250 bp) was performed at Azenta, Suzhou, China.

### **Metatranscriptomic sequencing**

1μg total RNA was used for following library preparation. The rRNA was depleted from the total RNA using rRNA removal Kit (Vazyme, Nanjing, China). The ribosomal depleted RNA was then fragmented and reverse-transcribed. First-strand cDNA was synthesized using ProtoScript II Reverse Transcriptase (Vazyme, Nanjing, China) with random primers and Actinomycin D. The second-strand cDNA was synthesized using Second Strand Synthesis Enzyme Mix (including dACG-TP/dUTP) (Vazyme, Nanjing, China). The purified double-stranded cDNA by beads was then treated with End Prep Enzyme Mix (Vazyme, Nanjing, China) to repair both ends and add a dA-tailing in one reaction, followed by a T-A ligation to add adaptors to both ends. Size selection of adaptor-ligated DNA was then performed using beads, and fragments of ~400 bp (with the approximate insert size of 300 bp) were recovered. The dUTP-marked second strand was digested with Uracil-Specific Excision Reagent enzyme (Vazyme, Nanjing, China). Each sample was then amplified by PCR using P5 and P7 primers, with both primers carrying sequences that can anneal to the flow cell to perform bridge PCR and P5/P7 primer carrying indices allowing for multiplexing. The libraries were pair-end sequenced (PE150) on the Illumina HiSeq X Ten System.

### **Fluorescence *in situ* hybridization (FISH)**

Enrichment samples were fixed with 4% paraformaldehyde for overnight at 4℃, washed three times with PBS, air dried, and dehydrated in 50%, 80%, and 100% ethanol for 3 min each at room temperature, then immobilized on the microscopic slides. Oligonucleotide probes were commercially synthesized and labeled with fluorescein isothiocyanate (FITC). The specimens were incubated with the FITC-labeled oligonucleotide probes at a concentration of 25 ng/μL of hybridization buffer [0.9 M NaCl, 20 mM Tris/HCl (pH 7.2), 30% formamide, 0.01% (w/v) SDS] at 46 °C for 4 h. After washing, the samples were submerged in cold ddH_2_O for a couple of seconds and air dried completely at ambient temperature. Samples were then stained with 4', 6-diamidino-2-phenylindole (DAPI, 2 μg/mL) (Thermo Fisher Scientific, Waltham, Massachusetts, USA). Finally, bright field and fluorescence images were acquired by DeltaVision Ultra microscopic imaging system (GE healthcare, Chicago, Illinois, USA).

### **DNA-SIP gradient fractionation**

3 µg of DNA from the extract was mixed with CsCl and loaded into a heat-sealable OpitSeal polypropylene tube (Beckman Coulter, Brea, California, USA) to obtain an initial buoyant density (BD) of 1.714 g mL^− 1^. The mixture was centrifuged at 408,500 g at 20 ^◦^C for 48 h using the Optima XPN-100 Ultracentrifuge (Beckman Coulter, Brea, California, USA). A fraction collector (Beckman Coulter, Brea, California, USA) was used to fractionate the CsCl into twenty-four equal volumes. The BD of each fraction was measured by a digital refractometer (Palette, ATAGO, Japan), and the fractions within the BD values range of 1.68-1.78 g mL^− 1^ were selected to purify and quantify the 16S rRNA gene copy numbers.

### **Quantitative PCR of 16S rRNA genes**

Quantification of total bacterial 16S rRNA across density fractions were assessed by quantitative PCR (qRCR) using primer sets 338F (5′-ACTCCTACGGGAGGCAGCAG-3′) / 518R (5′-ATTACCGCGGCTGCTGG-3′) with a thermal profile of 15 min at 95 ºC, followed by 45 cycles of 15 s at 94 ºC, 30 s at 55 ºC, and 30 s at 72 ºC. qPCR assays were carried out in a volume of 20 μL, containing 10 μL of Premix (TB Green; Takara, Japan), 0.2 μL of each primer, and 1 μL DNA template, and was topped up with ddH_2_O to a total volume of 20 μL. The standard curve was constructed from a series of tenfold dilutions of a known copy number of plasmid DNA. Negative controls, in which the DNA template was replaced by nuclease-free water, were also performed.

## **SUPPLEMENTARY RESULTS**

### **Nitrogen cycle in *Sysuimicrobiota***

The genes (*gdhA* and/or *gudB*) encoding for glutamate dehydrogenase were detected in 111 MAGs of *Sysuimicrobiota*, suggesting their ability to form glutamate from NH_3_ and 2-oxoglutarate. MAGs of *Sysuimicrobiaceae*, *Thermofontiviventaceae*, *Kaftiobacteriaceae* and *Humicultoraceae* contain nitrite reductase coding genes *nirK*, indicating they might have the ability to use nitrite as an electron acceptor for anaerobic respiration. MAGs in all families except *Segetimicrobiaceae* encode nitrite oxidoreductase (NxrAB)/nitrate reductase (NarBGHI) complex. Phylogenetic analysis suggested that NxrA/NarG genes recovered from *Sysuimicrobiota* are distributed in three clades (Fig. S19). NxrA/NarG genes from *Kaftiobacteriaceae* and *Humicultoraceae* were closely related to the known periplasmic NXRs of *Nitrospira*, *Nitrospina*, and anammox which indicates the potential capacity of nitrite oxidation to nitrate [5, 6]. NxrA/NarG genes from *Sysuimicrobiaceae* and *Thermofontiviventaceae* is grouped with the NxrA/NarG clade, which indicates the utilization of nitrate as an electron acceptor for anaerobic respiration. Meanwhile, nitrite reductase (*nirK*), nitric oxide reductase (*norB*), and nitrous-oxide reductase (*nosZ*) were present in MAGs of *Sysuimicrobiaceae*, suggesting MAGs of *Sysuimicrobiaceae* harbor gene sets for denitrification. *Sysuimicrobiaceae*, *Thermofontiviventaceae*, *Kaftiobacteriaceae* and *Humicultoraceae* harbored the potential for reducing the nitrous oxide to nitrogen due to the presence of the *nosZ* gene, which encodes nitrous-oxide reductase [7]. Genes involved in urea production were also found in *Sysuimicrobiota* MAGs, suggesting that *Sysuimicrobiota* may convert ammonium to urea.

### **Transcriptomic profile of nitrogen cycle**

The expression of NxrA/NarG genes confirmed the capacity of nitrate utilization as electron acceptor for *Sysuimicrobiacea*, and the capacity of nitrite oxidation to nitrate for *Kaftiobacteriaceae* and *Humicultoraceae* (Fig. 5B). In addition, nitrite reductase gene (*nirK*) for the response of nitrite reduction [8] were expressed, guaranteeing the capacity of nitrite utilization as electron acceptor for MAGs in *Sysuimicrobiota*. *Sysuimicrobiaceae*, *Kaftiobacteriaceae* and *Humicultoraceae* harbored a potential for reducing nitrous-oxide to nitrogen due to the detected expression of the *nosZ* gene. In total, gene expression profiles revealed that *Sysuimicrobiota* may play an important role in nitrogen cycle of hot spring.

### **The reconstruction of nitrogen and sulfur metabolism in the enriched treatment**

Besides *Sysuimicrobiota*, *Pseudomonadota*, and *Bacillota*, the rest of potential autotrophs were found in members of *Chloroflexota*, *Gemmatimonadota*, *Bacteroidota*, *Moduliflexota*, *Actinomycetota*, *Deinococcota,* and other phyla (Fig.4 and Table.S6). The enrichment also included lower abundances of heterotrophs, including members of the *Thermoproteota*, *Bipolaricaulota*, *Calescibacterota*, *Cyanobacteriota*, *Elusimicrobiota*, and *Hydrogenedentota*, they have organotrophic potential based on genes related to carbohydrate degradation, and no complete carbon fixation pathway could be detected in their genomes.

Nitrogen and sulfur cycling seem to be the main drivers of energetic processes in the enriched ecosystem. The presence of sufficient nitrite in the enrichment medium resulted in an abundance of genes related to nitrogen metabolism in the enriched community. Nitrous oxide can be produced from nitrite by *Chloroflexota*, *Pseudomonadota,* and *Bacillota*. The *Sysuimicrobiota* and co-cultured microbes can reduce the accumulated nitrous oxide by converting it to nitrogen. Then nitrogen can be further immobilized by *Bacillota* to generate ammonia. For sulfur cycling, *Sysuimicrobiota* can produce APS via sulfate adenylyltransferase, and the intermediates can be used by other co-cultures. Most microbes in the enriched micro ecosystem can reduce sulfate to H_2_S *via* assimilatory/dissimilatory sulfate reduction. In the genome of *Pseudomonadota*, genes related to dissimilatory sulfate oxidation have been identified, which indicated a potential transfer from H_2_S to sulfate.

## **NOMENCLATURE OF MEMBERS IN *SYSUIMICROBIOTA***

**Description of *Sysuimicrobiota* phy. nov.**

*Sysuimicrobiota* (Sy.su.i.mic.ro’bi.o’ta. N.L. neut. n. *Sysuimicrobium* type genus of the phylum; L. suff. –*ota* ending to denote a phylum; N.L. pl. neut. n. *Sysuimicrobiota* the *Sysuimicrobium* phylum).

Type genus: *Sysuimicrobium*.

**Description of *Sysuimicrobiia* class nov.**

*Sysuimicrobiia* (Sy.su.i.mic.ro’bi.ia. N.L. neut. n. *Sysuimicrobium* fererring to the type genus of the class; L. suff. –*ia* ending to denote a class; N.L. neut. pl. n. *Sysuimicrobiia* the *Sysuimicrobium* class).

The description of the class is the same as for the order *Sysuimicrobiales*.

Type order: *Sysuimicrobiales*.

**Description of *Sysuimicrobiales* ord. nov.**

*Sysuimicrobiales* (Sy.su.i.mi.cro’bi.a’les. N.L. neut. n. *Sysuimicrobium* type genus of the order; L. suff. –*ales* ending to denote an order; N.L. fem. pl. n. *Sysuimicrobiales* the *Sysuimicrobium* order).

The order *Sysuimicrobiales* comprised of five families *Sysuimicrobiaceae* fam. nov., *Thermofontiviventaceae* fam. nov., *Segetimicrobiaceae* fam. nov., *Kaftiobacteriaceae* fam. nov. and *Humicultoraceae* fam. nov.

Type family: *Sysuimicrobiaceae*.

**Description of *Sysuimicrobiaceae* fam. nov.**

*Sysuimicrobiaceae* (Sy.su.i.mi.cro.bi.a.ce’ae. N.L. neut. n. *Sysuimicrobium* type genus of the family; L. suff. –*aceae* ending to denote a family; N.L. fem. pl. n. *Sysuimicrobiaceae* the *Sysuimicrobium* family).

The family at present contains three genera *Sysuimicrobium* gen. nov., *Caldifonticola* gen. nov. and *Tepidifontimicrobium* gen. nov.

Type genus: *Sysuimicrobium*.

**Description of *Sysuimicrobium* gen. nov.**

*Sysuimicrobium* (Sy.su.i.mi.cro.bi.um. N.L. neut. n. *microbium* microbe; N.L. neut. n. *Sysuimicrobium*, arbitrary name from the acronym of Sun Yat-Sen University, SYSU where the identification of microbial metagenome was done).

Type species: *Sysuimicrobium calidum*.

**Description of *Ca*. Sysuimicrobium thermophilum sp. nov.**

*Sysuimicrobium* *thermophilum* (ther.mo’phi.lum. Gr. fem. adj. *thermê* heat; N.L. neut. adj. suff. *-philum* loving, N.L. neut. adj. *thermophilum* heat loving).

Type material: Firmicutes_bacterium_SpSt-238, obtained from the metagenome assembly of a hot spring sediment sample from British Columbia, Canada.

**Description of *Sysuimicrobium* *tengchongense* sp. nov.**

*Sysuimicrobium* *tengchongense* (teng.cong.en’se. N.L. neut. adj. *tengchongense* referring to Tengchong).

Type material: BF2_201808_bins_94, obtained from the metagenome assembly of a hot spring sample from Tengchong, P.R. China.

**Description of *Sysuimicrobium* *calidum* sp. nov.**

*Sysuimicrobium* *calidum* (ca’li.dum. L. neut. adj. *calidum* indicating the origin of the metagenome from hot spring).

Type material: HHBFW-2_201803_bins_120, obtained from the metagenome assembly of a hot spring sample from Tengchong, P.R. China.

**Description of *Caldifonticola* gen. nov.**

*Caldifonticola* (Cal.di.fon.ti’co.la. L. adj. *caldus* hot; L. masc. n. *fons* a spring; N.L. masc. suff. -*cola* an inhabitant; N.L. masc. n. *Caldifonticola* inhabitant of a hot spring).

Type species: *Caldifonticola* *tengchongensis*.

**Description of *Caldifonticola* *tengchongensis* sp. nov.**

*Caldifonticola* *tengchongensis* (teng.chong.en’sis. N.L. masc. adj. *tengchongensis* pertaining to Tengchong, the source of the metagenomic sample).

Type material: BF3_201808_bins_64, obtained from a metagenome assembly of a hot spring sample from Tengchong, P.R. China.

**Description of *Tepidifontimicrobium* gen. nov.**

*Tepidifontimicrobium* (Te.pi.di.fon.ti.mi.cro’bi.um. L. masc. adj. *tepidus* moderately warm; L. masc. n. *fons* a spring; N.L. neut. n. *microbium* microbe; N.L. neut. n. *Tepidifontimicrobium* a microbe from a warm spring).

Type species: *Tepidifontimicrobium* *thermophilum*.

**Description of *Tepidifontimicrobium* *thermophilum* sp. nov.**

*Tepidifontimicrobium* *thermophilum* (ther.mo’phi.lum. Gr. fem. adj. *thermê* heat; N.L. neut. adj. suff. *-philum* loving, N.L. neut. adj. *thermophilum* heat loving).

Type material: HHBFW-2_201803_bins_14, obtained from a metagenome assembly of hot spring samples from Tengchong, P.R. China.

**Description of *Thermofontiviventaceae* fam. nov.**

*Thermofontiviventaceae* (Ther.mo.fon.ti.vi.ven.ta.ce’ae. N.L. masc. n. *Thermofontivivens* referring to the type genus *Thermofontivivens*; L. suff. –*aceae* ending to denote a family; N.L. fem. pl. n. *Thermofontivivantaceae* the *Thermofontivivens* family).

Type genus: *Thermofontivivens*.

**Description of *Thermofontivivens* gen. nov.**

*Thermofontivivens* (Ther.mo.fon.ti.vi’vens. Gr. masc. adj. *thermos* hot; L. masc. n. *fons* a spring; L. pres. part. *vivens* living; N.L. masc. n. *Thermofontivivens* living in a hot spring).

Type species: *Thermofontivivens* *primus*.

**Description of *Thermofontivivens* *primus* sp. nov.**

*Thermofontivivens* *primus* (pri’mus. L. masc. adj. *primus* first, to represent the first genome bin from of the genus).

Type material: QQ_201705_bins_113, obtained from the metagenome assembly of a hot spring sample from Tengchong, P.R. China.

**Description of *Ca*. Thermofontivivens secundus sp. nov.**

*Thermofontivivens* *secundus* (se.cun’dus. L. masc. adj. *secundus* second, to represent the second genome bin of the genus).

Type material: Firmicutes_bacterium_SpSt-165, obtained from the metagenome assembly of a hot spring sediment sample from British Columbia, Canada.

**Description of *Segetimicrobiaceae* fam. nov.**

*Segetimicrobiaceae* (Se.ge’ti.mi.cro.bi.a.ce’ae. N.L. neut. n. *Segetimicrobium* type genus of the family; L. suff. –*aceae* ending to denote a family; N.L. fem. pl. n. *Segetimicrobiaceae*

the *Segetimicrobium* family).

Type genus: *Segetimicrobium*.

**Description of *Segetimicrobium* gen. nov.**

*Segetimicrobium* (Se.ge’ti.mi.cro’bi.um. L. gen. n. *segetis* of the soil; N.L. neut. n. *microbium* microbe; N.L. neut. n. *Segetimicrobium* a microbe of the earth).

Type species: *Segetimicrobium genomatis*.

**Description of *Ca*. Segetimicrobium genomatis sp. nov.**

*Segetimicrobium genomatis* (ge.no.ma’tis. N.L. gen. n. *genomatis* of a genome).

Type material: Terrabacteria_group_bacterium_ANGP1_NP_3, obtained from the metagenome assembly from a temperate grassland biome from Angelo Coast Range Reserve, California, USA.

**Description of *Kaftiobacteriaceae* fam. nov.**

*Kaftiobacteriaceae* (Kaf.ti.o.bac.te.ri.a.ce’ae. N.L. neut. n. *Kaftiobacterium* type genus of the family; L. suff. –*aceae* ending to denote a family; N.L. fem. pl. n. *Kaftiobacteriaceae* the *Kaftiobacterium* family).

The family at present contains three genera *Kaftiobacterium* gen. nov., *Ca*. Telluricultor gen. nov. and *Calidihabitans* gen. nov.

Type genus: *Kaftiobacterium*.

**Description of *Kaftiobacterium* gen. nov.**

*Kaftiobacterium* (Kaf.ti.o.bac.te.ri.um. Gr. adj. *kafti* hot; N.L. neut. n. *bacterium* rod or staff and, in biology a bacterium; N.L. neut. n. *Kaftiobacterium* a bacterium in hot environment).

Type species: *Kaftiobacterium* *secundum*.

**Description of *Ca*. Kaftiobacterium primum sp. nov.**

*Kaftiobacterium* *primum* (pri’mum. L. neut. adj. *primum* first, to represent the first genome bin from of the genus).

Type material: Firmicutes_bacterium_SpSt-351, obtained from the metagenome assembly of a hot spring sediment sample from British Columbia, Canada.

**Description of *Kaytiobacterium* *secundum* sp. nov.**

*Kaytiobacterium* *secundum* (se.cun’dum. L. neut. adj. *secundum* second, to represent the second genome bin of the genus).

Type material: JZ-2_201705_bins_188, obtained from the metagenome assembly of a hot spring sample from Tengchong, P.R. China.

**Description of *Ca*. Telluricultor gen. nov.**

*Telluricultor* (Tel.lu.ri.cul’tor. L. gen. n. *telluris* of the soil or earth; L. masc. n. *cultor* an inhabitant; N.L. masc. n. *Telluricultor* an inhabitant of the soil).

Type species: *Ca.* Telluricultor primus.

**Description of *Ca.* Telluricultor primus sp. nov.**

*Telluricultor* *primus* (pri’mus. L. masc. adj. *primus* first, to represent the first genome bin from of the genus).

Type material: Terrabacteria_group_bacterium_ANGP1_NP_4, obtained from the metagenome assembly from a temperate grassland biome from Angelo Coast Range Reserve, California, USA.

**Description of *Ca.* Telluricultor secundus sp. nov.**

*Telluricultor* *secundus* (se.cun’dus. L. masc. adj. *secundus* second, to represent the second genome bin of the genus).

Type material: Terrabacteria_group_bacterium_ANGP1_NP_8, obtained from the metagenome assembly from a temperate grassland biome from Angelo Coast Range Reserve, California, USA.

**Description of *Calidihabitans* gen. nov.**

*Calidihabitans* (Ca.li.di.ha.bi.tans. L. masc. adj. *calidus* hot; L. pres. part. *habitans* inhabiting; N.L. masc. n. *Calidihabitans* an inhabitant of hot environment).

Type species: *Calidihabitans* *tengchongensis*.

**Description of *Calidihabitans* *tengchongensis* sp. nov.**

*Calidihabitans* *tengchongensis* (teng.chong.en’sis. N.L. masc. adj. *tengchongensis* pertaining to Tengchong, the source of the metagenomic sample).

Type material: JZ-2_201709_bins_171, obtained from a metagenome assembly of a hot spring sample from Tengchong, P.R. China.

**Description of *Humicultoraceae* fam. nov.**

*Humicultoraceae* (Hu.mi.cul.tor.a.ce’ae. N.L. masc. n. *Humicultor* type genus of the family; L. suff. –*aceae* ending to denote a family; N.L. fem. pl. n. *Humicultoraceae* the *Humicultor* family).

The family at present contains three genera *Humicultor* gen. nov., *Geohabitans* gen. nov. and *Fervidifonticultor* gen. nov.

Type genus: *Humicultor*.

**Description of *Humicultor* gen. nov.**

*Humicultor* (Hu.mi.cul’tor. L. fem. n. *humus* soil; L. masc. n. *cultor* an inhabitant; N.L. masc. n. *Humicultor* an inhabitant of soil).

Type species: *Humicultor* *tengchongensis*.

**Description of *Ca.* Humicultor riflensis sp. nov.**

*Humicultor* *riflensis* (rifl.en’sis. L. masc. adj. *riflensis* referring to Rifle).

Type material: Armantimonadetes_bacterium_CSP1-3, obtained from the metagenome assembly of a sediment sample from Rifle, Colorado, USA.

**Description of *Humicultor* *tengchongensis* sp. nov.**

*Humicultor* *tengchongensis* (teng.chong.en’sis. N.L. masc. adj. *tengchongensis* pertaining to Tengchong, the source of the metagenomic sample).

Type material: JZ-2_201709_bins_123, obtained from a metagenome assembly of a hot spring sample from Tengchong, P.R. China.

**Description of *Geohabitans* gen. nov.**

*Geohabitans* (Ge.o.ha.bi.tans. Gr. n. *geo*, the earth; L. pres. part. *habitans*, inhabiting; N.L. masc. n. *Geohabitans*, an inhabitant of Earth).

Type species: *Geohabitans* *tengchongensis.*

**Description of *Ca.* Geohabitans canadensis sp. nov.**

*Geohabitans* *canadensis* (can.den’sis. N.L. masc. adj. *canadensis* pertaining to Canada, the country from where the genome is extracted).

Type material: Firmicutes_bacterium_SpSt-190, obtained from the metagenome assembly of a hot spring sediment sample from British Columbia, Canada.

**Description of *Geohabitans* *tengchongensis* sp. nov.**

*Geohabitans tengchongensis* (teng.chong.en’sis. N.L. masc. adj. *tengchongensis* pertaining to Tengchong, the source of the metagenomic sample).

Type material: JZ-2_201803_bins_100, obtained from a metagenome assembly of a hot spring sample from Tengchong, P.R. China.

**Description of *Fervidifonticultor* gen. nov.**

*Fervidifonticultor* (Fer.vi.di.fon.ti.cul’tor. L. masc. adj. *fervidus* hot, burning; L. masc. n. *fons* a spring, fountain; L. masc. n. *cultor* an inhabitant; N.L. masc. n. *Fervidifonticultor* inhabitant of a hot spring).

Type species: *Fervidifonticultor* *primus*

**Description of *Fervidifonticultor* *primus* sp. nov.**

*Fervidifonticultor* *primus* (pri’mus. L. masc. adj. *primus* first, to represent the first genome bin from of the genus).

Type material: HHBFW-2_201803_bins_245, obtained from a metagenome assembly of a hot spring sample from Tengchong, P.R. China.

**Description of *Ca.* Fervidifonticultor secundus sp. nov.**

*Fervidifonticultor* *secundus* (se.cun’dus. L. masc. adj. *secundus* second, to represent the second genome bin of the genus).

Type material: JZ-2_201808_bins_221, obtained from a metagenome assembly of a hot spring sample from Tengchong, P.R. China.

**Description of *Ca.* Fervidifonticultor tertius sp. nov.**

*Fervidifonticultor* *tertius* (ter’ti.us. L. masc. adj. *tertius* third, to represent the third genome bin of the genus).

Type material: JZ-2_201907_bins_90, obtained from a metagenome assembly of a hot spring sample from Tengchong, P.R. China.

**Description of *Ca.* Fervidifonticultor quartus sp. nov.**

*Fervidifonticultor* *quartus* (qu.ar’tus. L. masc. adj. *quartus* fourth, to represent the fourth genome bin of the genus).

Type material: JZ-4_201709_bins_106, obtained from a metagenome assembly of a hot spring sample from Tengchong, P.R. China.

**Description of *Ca.* Fervidifonticultor quintus sp. nov.**

*Fervidifonticultor* *quintus* (qu.in’tus. L. masc. adj. *quintus* fifth, to represent the fifth genome bin of the genus).

Type material: JZ-2_201803_bins_44, obtained from a metagenome assembly of a hot spring sample from Tengchong, P.R. China.

**Description of *Ca.* Fervidifonticultor sextus sp. nov.**

*Fervidifonticultor* *sextus* (sex’tus. L. masc. adj. *sextus* sixth, to represent the sixth genome bin of the genus).

Type material: HHBFW-2_201803_bins_99, obtained from a metagenome assembly of a hot spring sample from Tengchong, P.R. China.

**Description of *Ca.* Fervidifonticultor septimus sp. nov.**

*Fervidifonticultor* *septimus* (sep.ti’mus. L. masc. adj. *septimus* seventh, to represent the seventh genome bin of the genus).

Type material: JZ-2_201808_bins_132, obtained from a metagenome assembly of a hot spring sample from Tengchong, P.R. China.

**Description of *Ca.* Fervidifonticultor octavus sp. nov.**

*Fervidifonticultor* *octavus* (oc.ta’vus. L. masc. adj. *octavus* eighth, to represent the eighth genome bin of the genus).

Type material: JZ-2_201912_bins_183, obtained from a metagenome assembly of a hot spring sample from Tengchong, P.R. China.

# **SUPPLEMENTARY FIGURES**

**
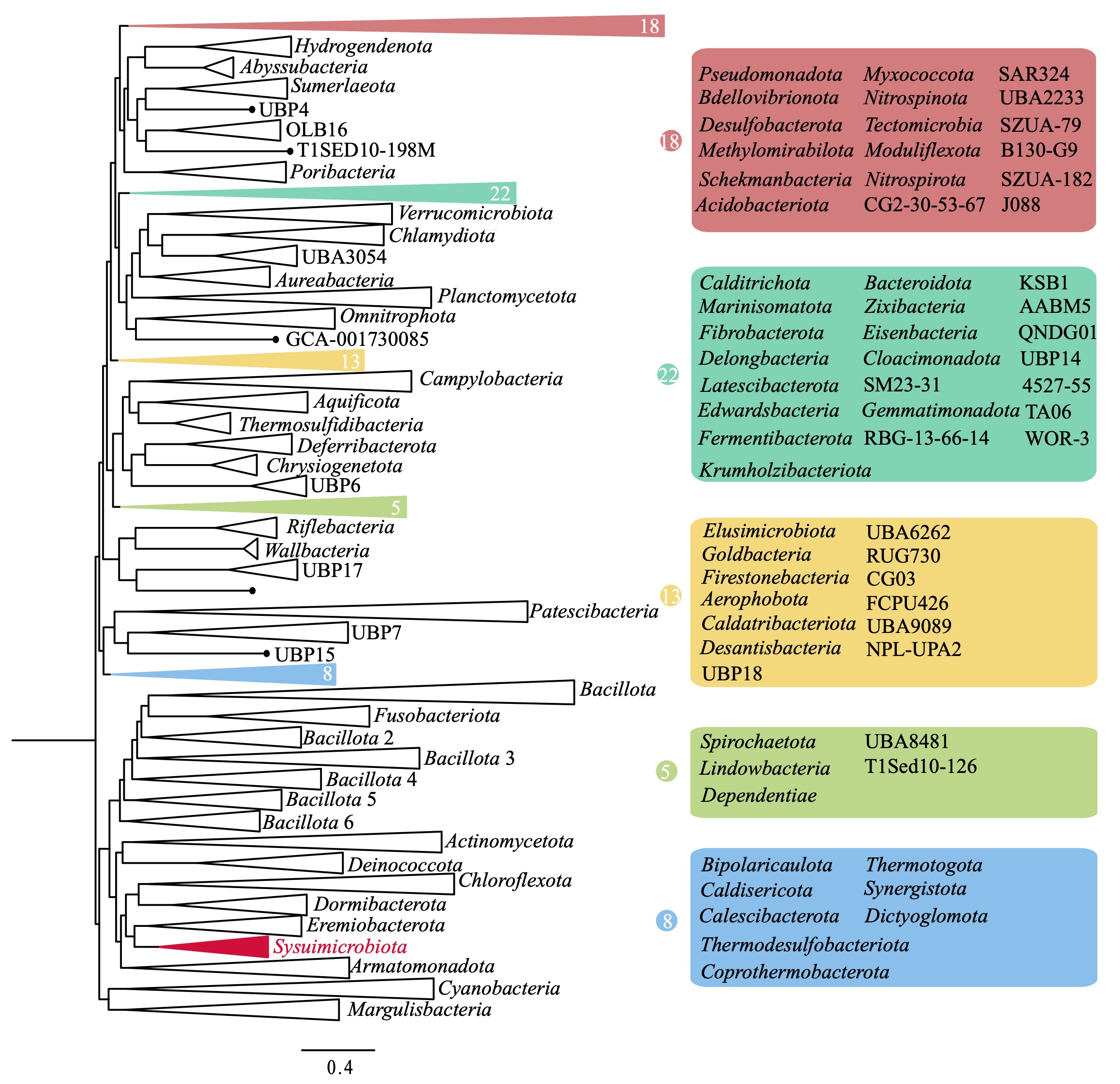
**

**Supplementary Fig. S1. Phylogenetic tree showing the relationships of *Sysuimicrobiota* and other phyla.** Multiple sequence alignments of 120 marker genes generated by GTDB-Tk were used to construct the phylogenetic tree by FastTree with the model LG+F+R10.

**
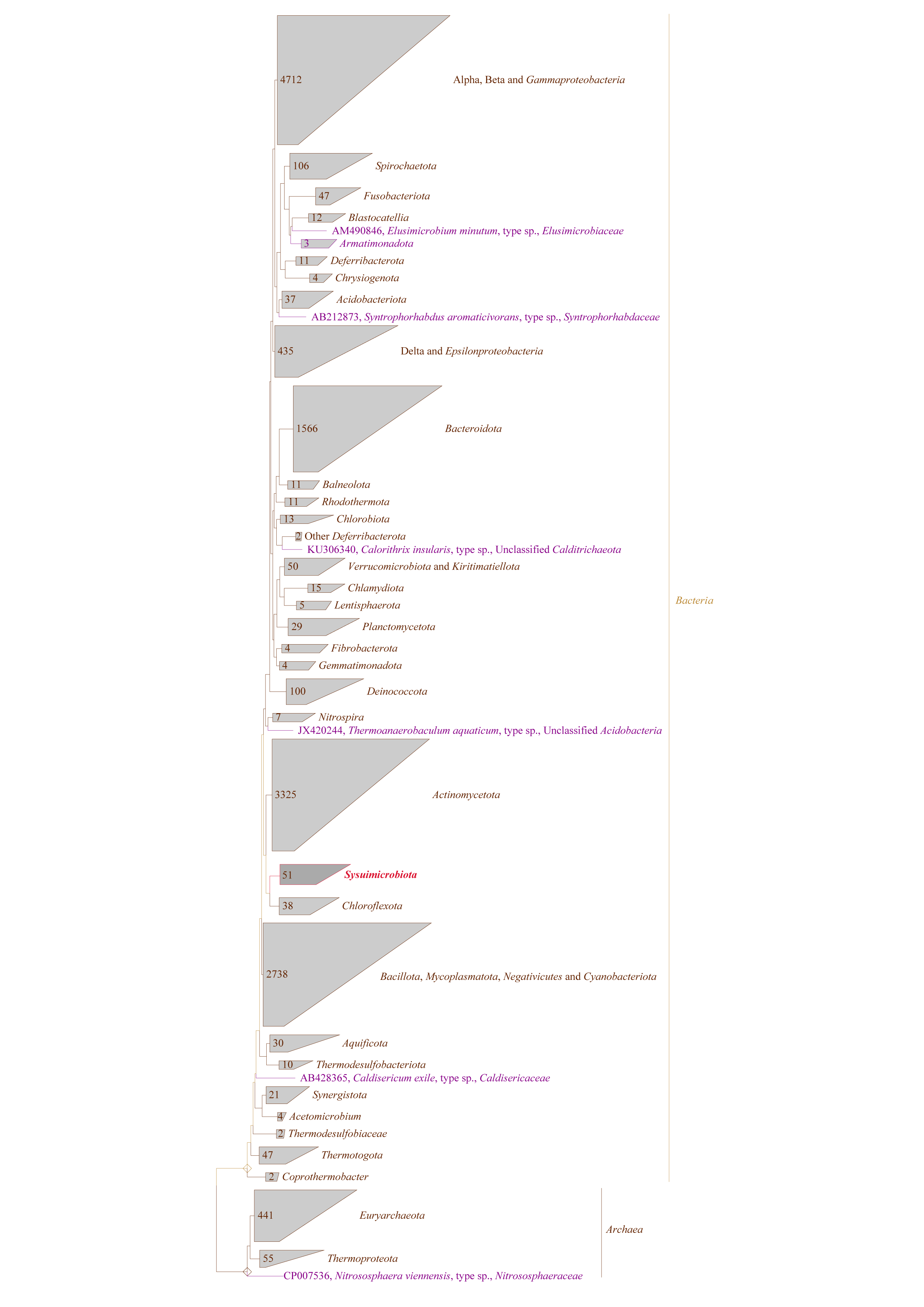
**

**Supplementary Fig. S2. The phylogenetic tree of 16S rRNA gene sequences.** The 16S rRNA gene sequences were identified from the *Sysuimicrobiota* MAGs, while reference sequences were from the pre-aligned SILVA tree (‘LTPs132_SSU’ [9]) in ARB [10].

**
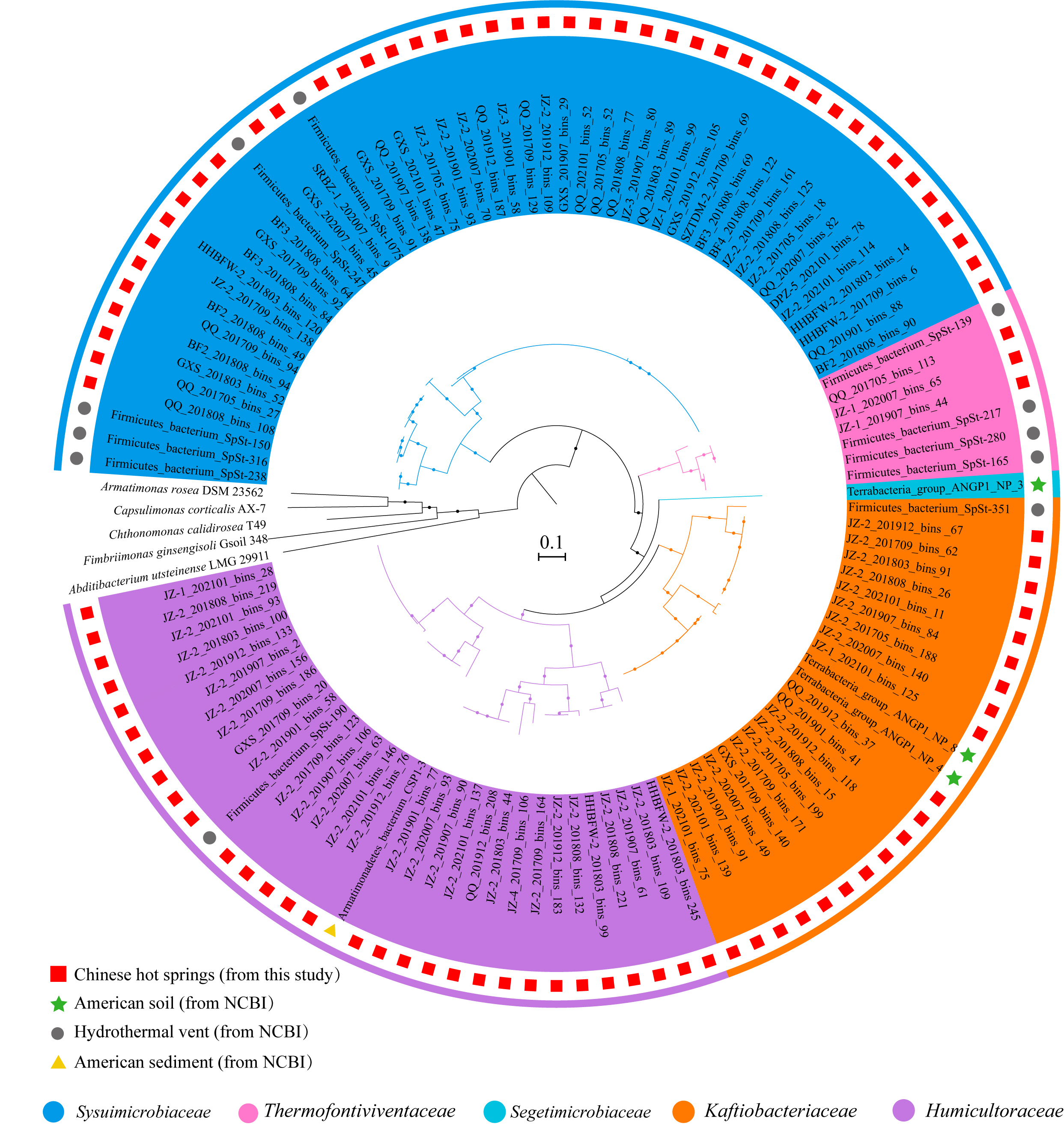
**

**Supplementary Fig. S3. Phylogenetic placements of *Sysuimicrobiota* MAGs based on 16 ribosomal proteins.** Sequences were aligned using MUSCLE5 [11], and divergent regions were eliminated using TrimAL[12]. The IQ-Tree was used for phylogenetic inference [13], and the best-fit model LG+F+R4 was well supported by Bayesian Information Criterion (BIC). Phylogenetic tree was visualized and annotated using iTOL [14]. Bootstrap values were based on 1000 replicates, and nodes with confidence > 70% are indicated as circles.

**Supplementary Fig. S4. Average amino acid identity (AAI) shared among genomes of *Sysuimicrobiota*.** To calculate the AAI of each pair of genomes, their predicted amino acid sequences were used to identify the orthologous genes based on reciprocal best BLAST hits, and the mean similarity of all orthologous genes was calculated as the AAI value.

**Supplementary Fig. S5. Average amino nucleotide identity (ANI) shared among genomes of *Sysuimicrobiota*.** ANI values were calculated by using the pyANI [15].

**
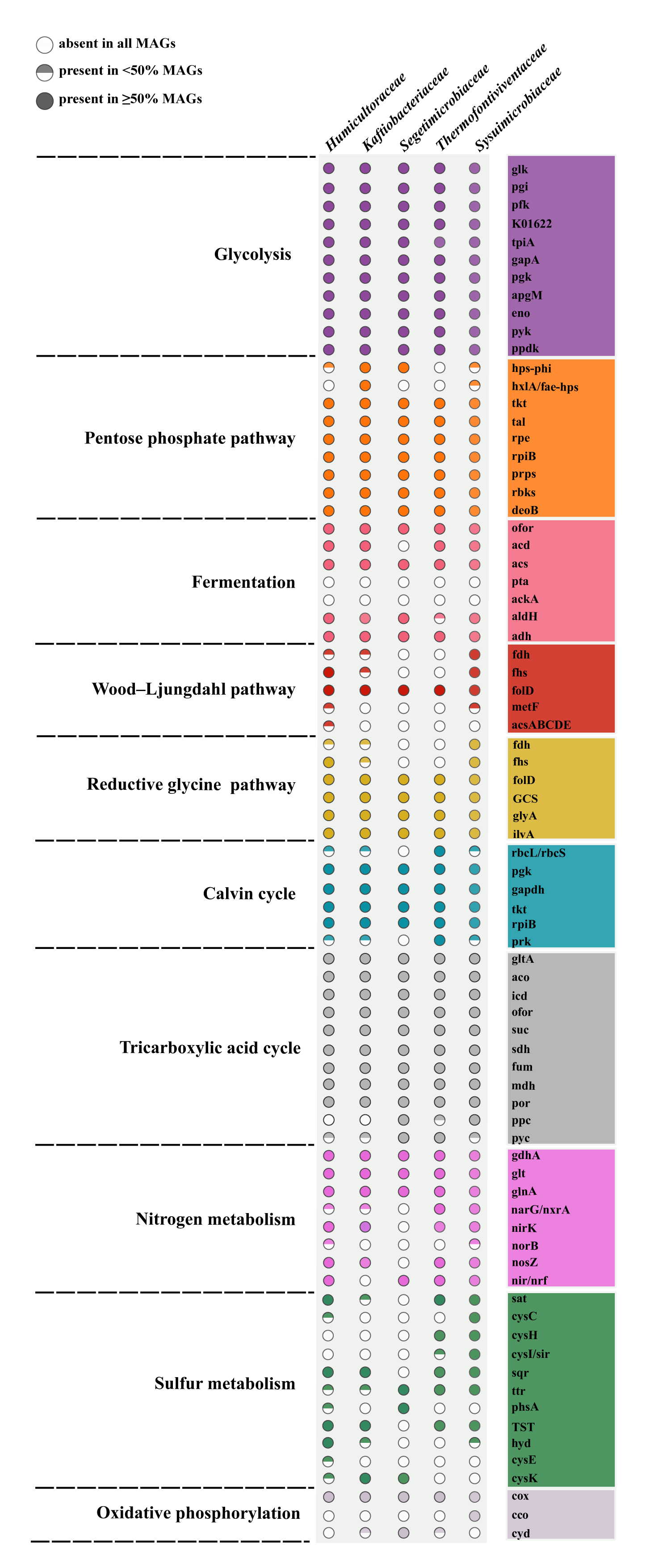
**

**Supplementary Fig. S6. The core metabolic pathways with the presence/absence of genes of the five novel families.** Hollow circles represent genes or metabolic pathways that were absent in all MAGs, solid circles represent genes or metabolic pathways that were present in ≥ 50% MAGs, and half-filled circles represent genes or metabolic pathways that were present in < 50% MAGs. Distinct metabolic pathways were distinguished by different colored dots. See Table S5 in the supplemental material for details.

**
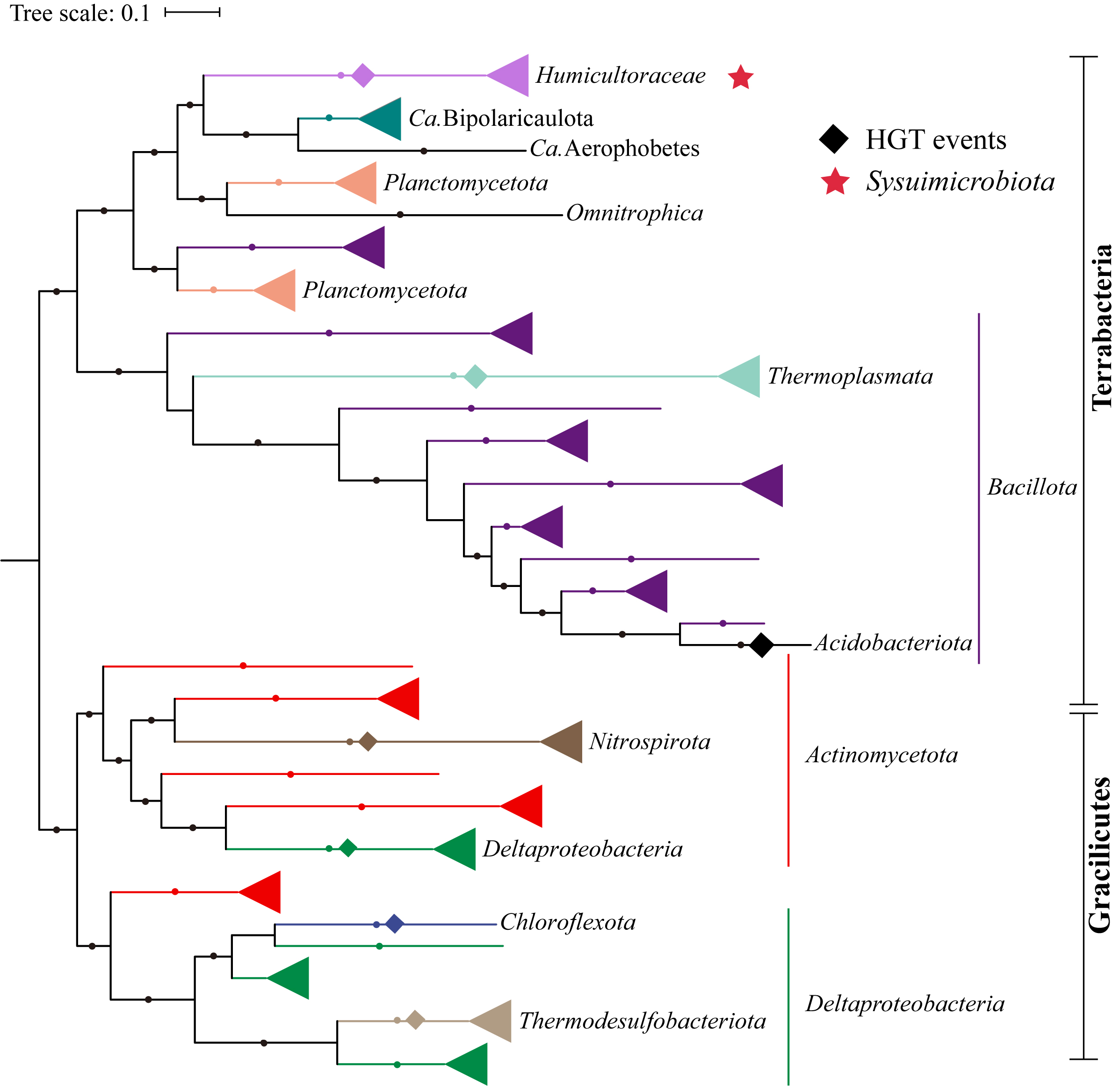
**

**Supplementary Fig. S7. Maximum-likelihood phylogeny of concatenated AcsDABCE.** The phylogenetic tree was rooted according to previous studies [16, 17]. Phylogenetic groups are colored. The clades are labeled with “Terrabacteria” and “Gracilicutes” according to a previous study [16].

**
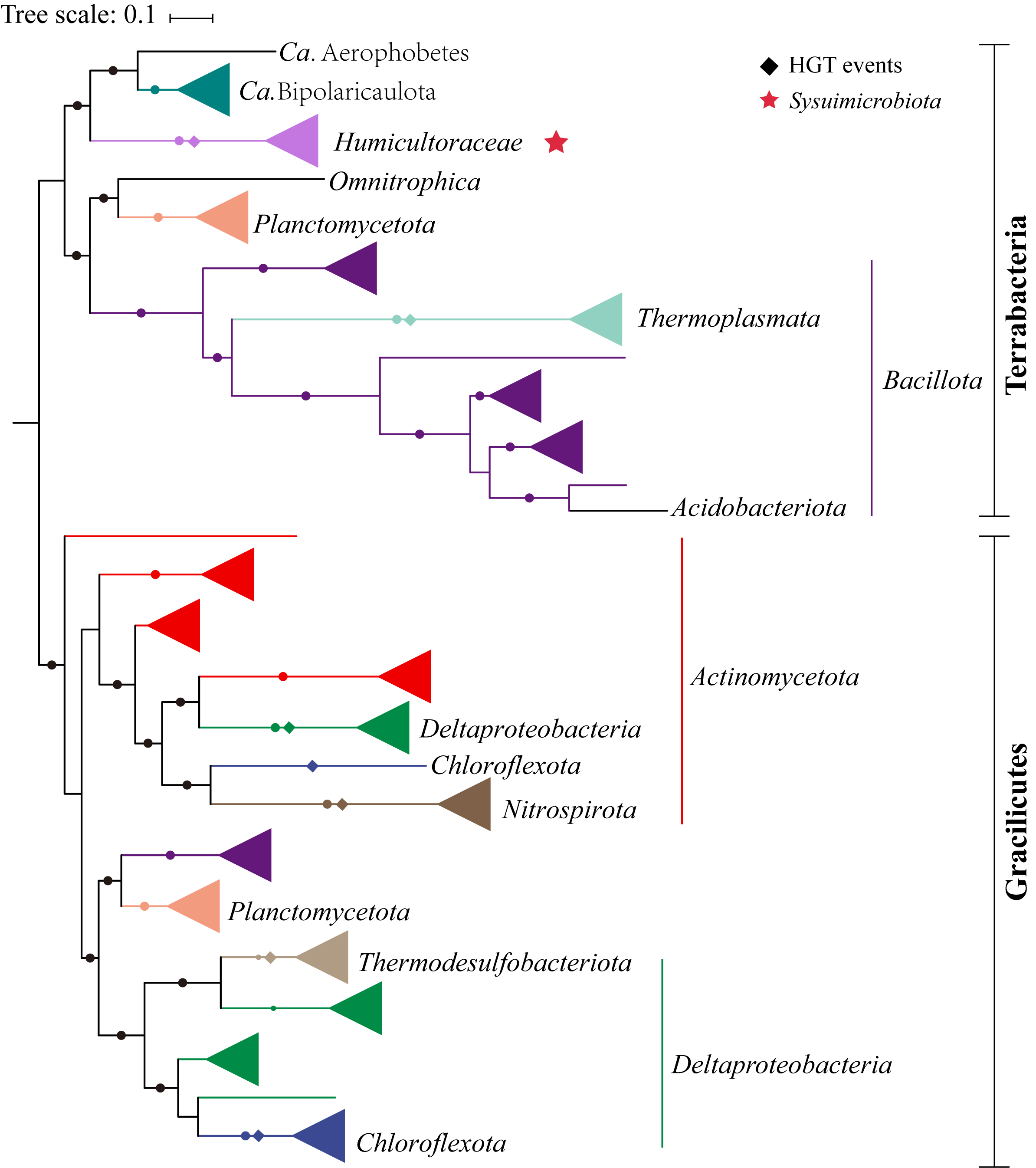
**

**Supplementary Fig. S8. Maximum-likelihood phylogeny of concatenated AcsAB.** The phylogenetic tree was rooted according to previous studies [16, 17]. Phylogenetic groups are colored. The clades are labeled with “Terrabacteria” and “Gracilicutes” according to a previous study [16].

**
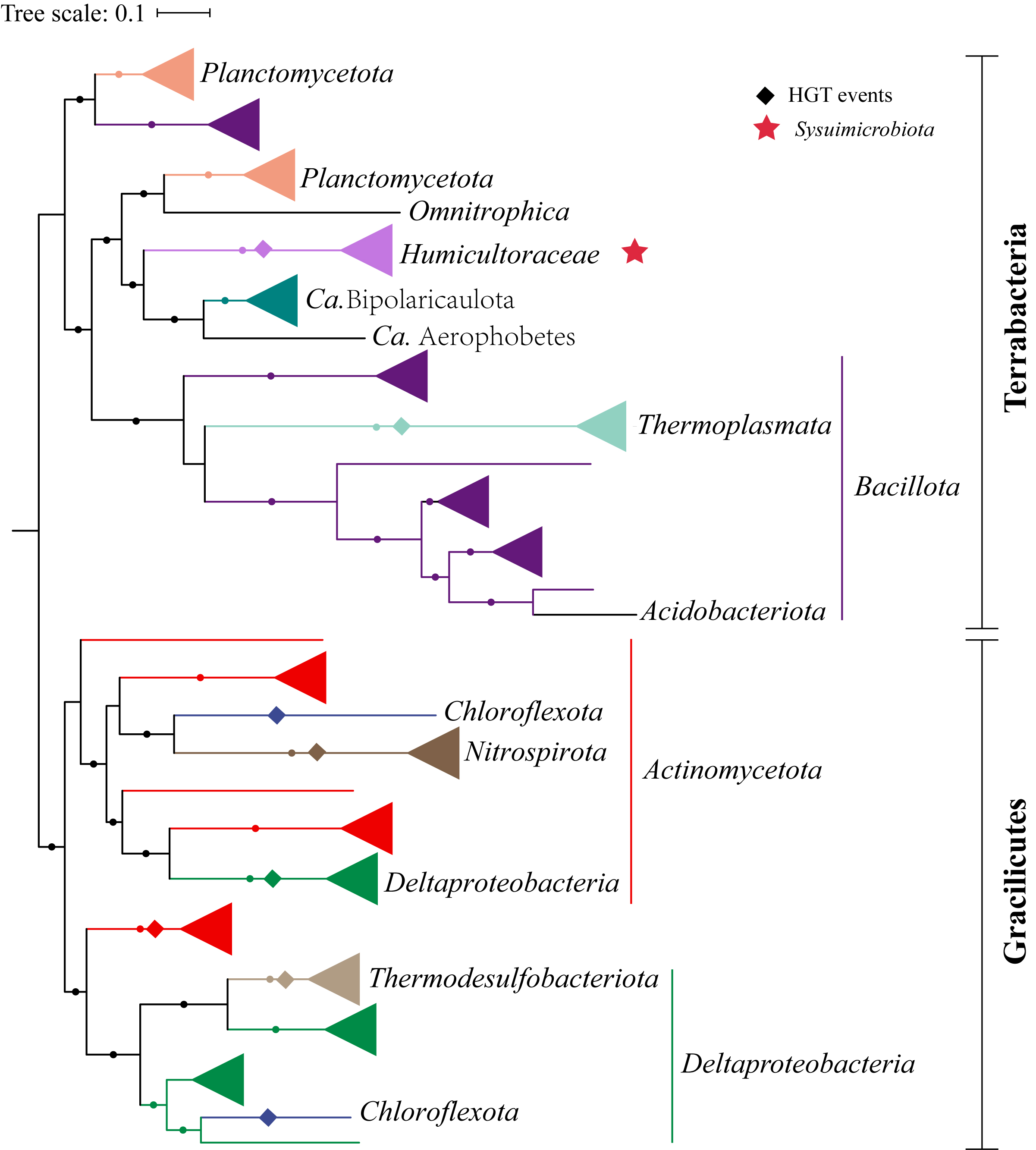
**

**Supplementary Fig. S9. Maximum-likelihood phylogeny of concatenated AcsABC.** The phylogenetic tree was rooted according to previous studies [16, 17]. Phylogenetic groups are colored. The clades are labeled with “Terrabacteria” and “Gracilicutes” according to a previous study [16].

**
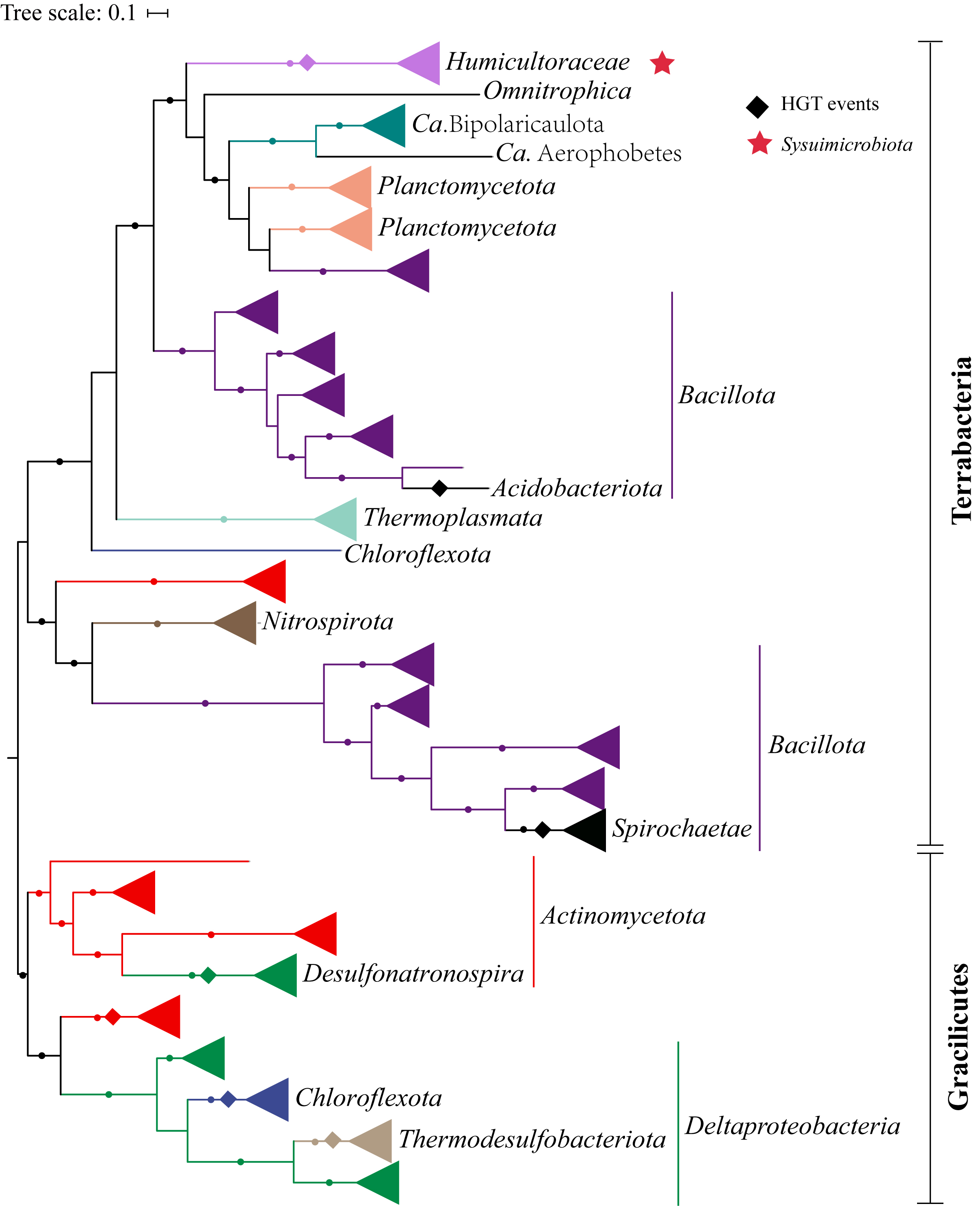
**

**Supplementary Fig. S10. Maximum likelihood phylogeny of concatenated AcsDEC.** The phylogenetic tree was rooted according to previous studies [16, 17]. Phylogenetic groups are colored. The clades are labeled with “Terrabacteria” and “Gracilicutes” according to a previous study [16].


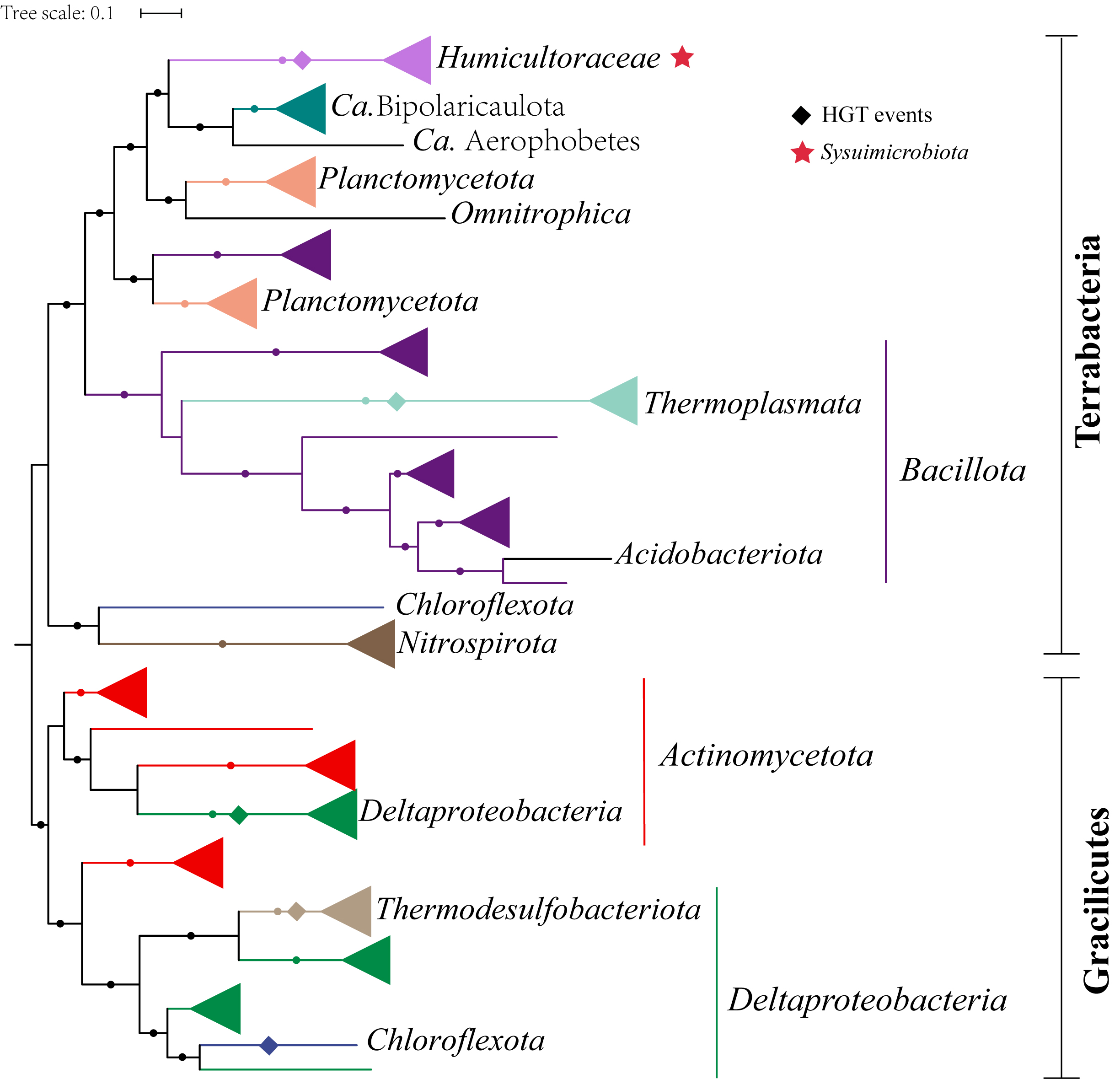


**Supplementary Fig. S11. Maximum likelihood phylogeny of concatenated AcsDABC.** The phylogenetic tree was rooted according to previous studies [16, 17]. Phylogenetic groups are colored. The clades are labeled with “Terrabacteria” and “Gracilicutes” according to a previous study [16].

**
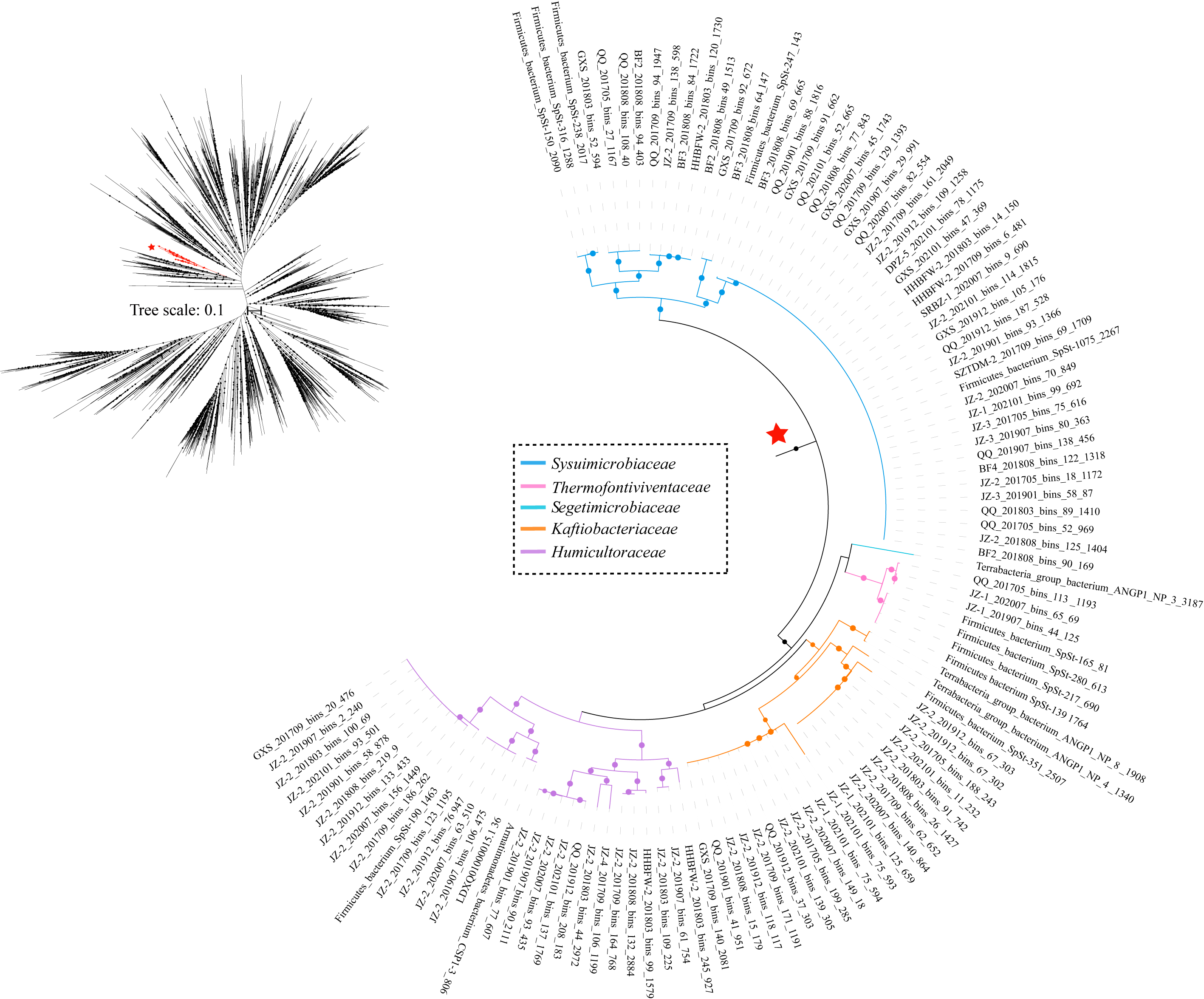
**

**Supplementary Fig. S12. Phylogenetic tree of GcvPA protein sequences.** The GcvPA sequences were aligned using MUSCLE5 [11], and divergent regions were eliminated using TrimAL [12]. The IQ-Tree was used for phylogenetic inference [13], and the best model LG+F+R10 was well supported by Bayesian Information Criterion (BIC). Phylogenetic tree was visualized and annotated using iTOL [14].


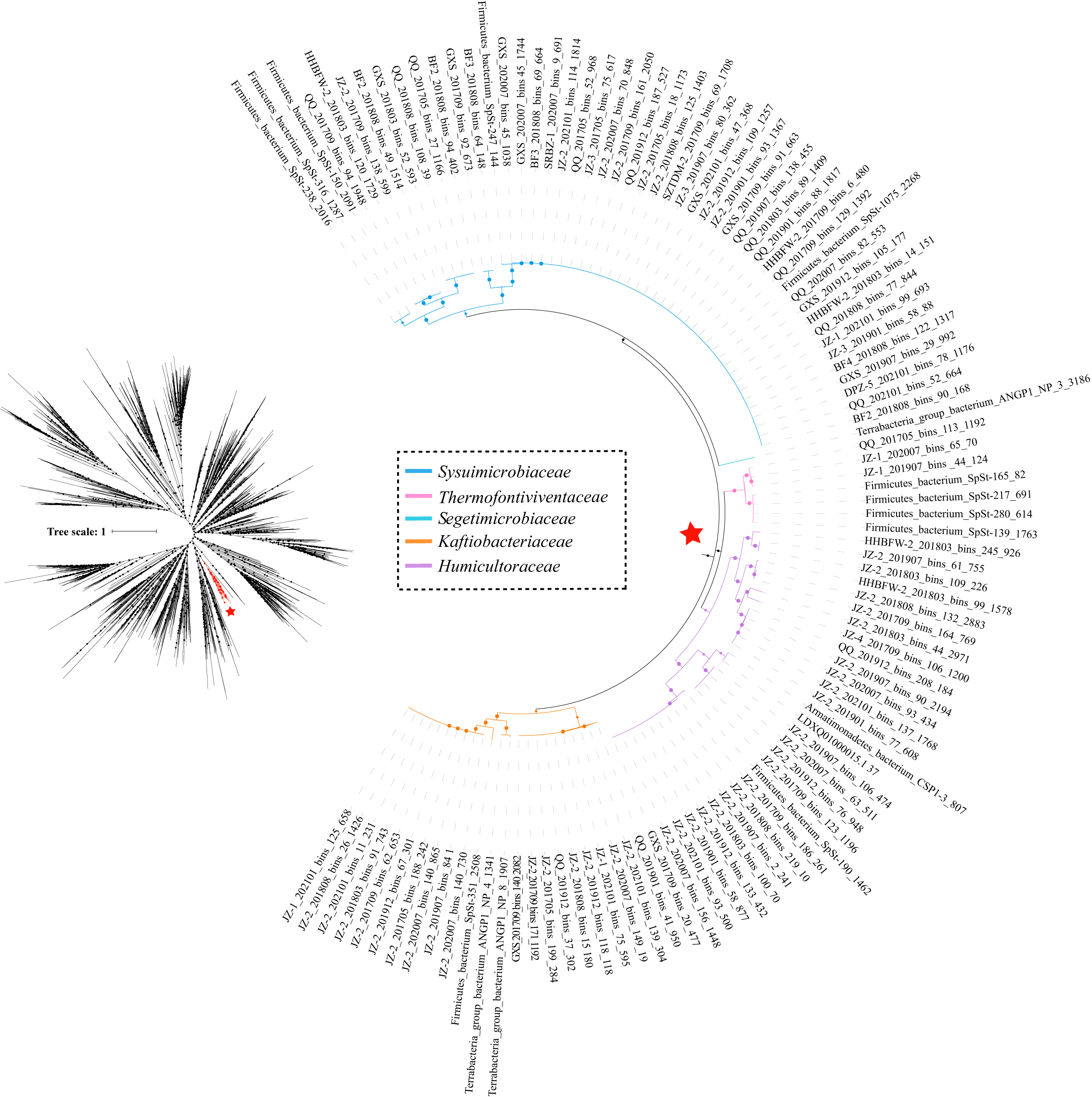


**Supplementary Fig. S13. Phylogenetic tree of GcvPB protein sequences.** The GcvPB sequences were aligned using MUSCLE5 [11], and divergent regions were eliminated using TrimAL[12]. The IQ-Tree was used for phylogenetic inference [13], and the best model LG+R10 was well supported by Bayesian Information Criterion (BIC). Phylogenetic tree was visualized and annotated using iTOL [14].

**
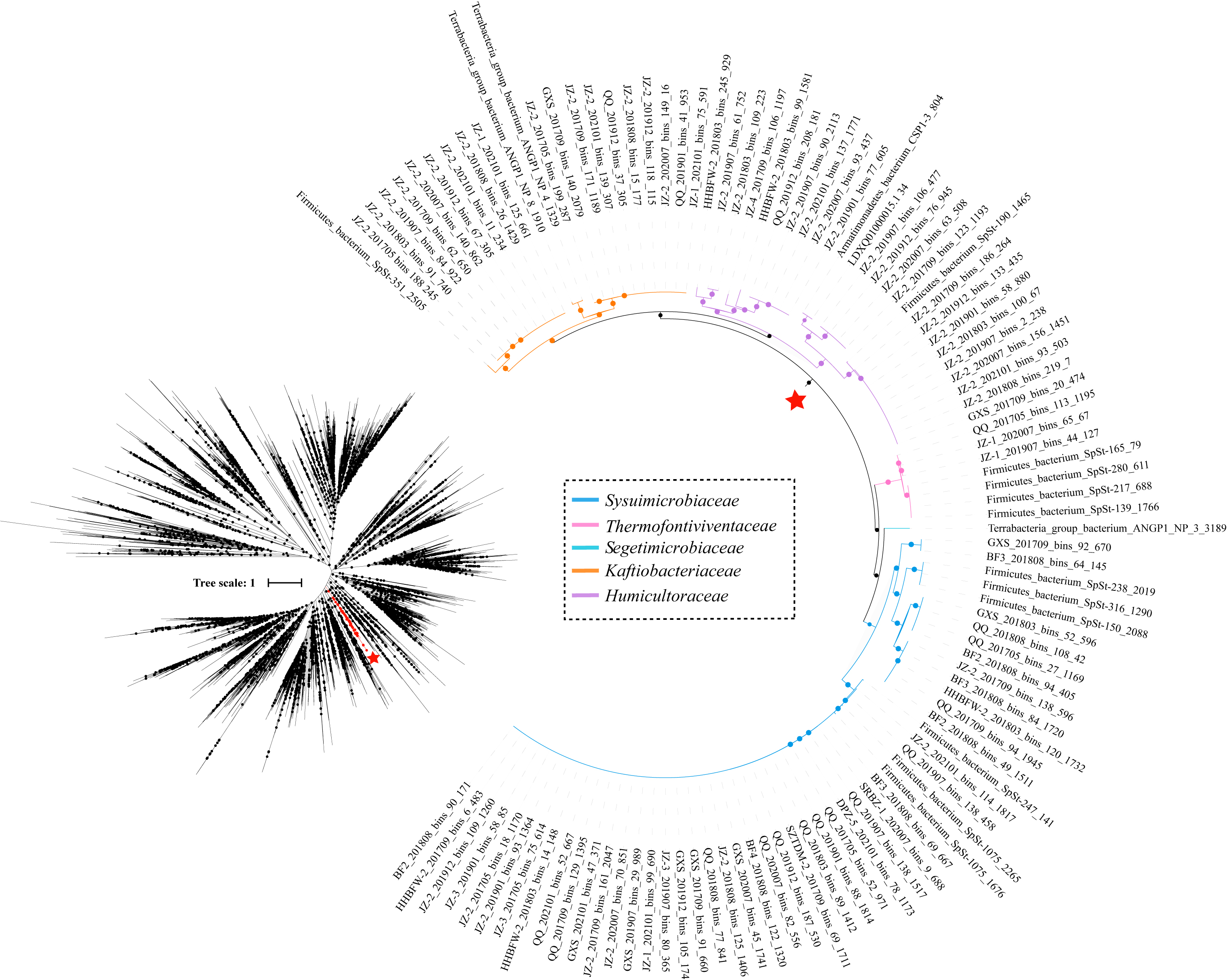
**

**Supplementary Fig. S14. Phylogenetic tree of GcvT protein sequences.** The GcvT sequences were aligned using MUSCLE5 [11], and divergent regions were eliminated using TrimAL [12]. The IQ-Tree was used for phylogenetic inference [13], and the best model LG+R10 was well supported by Bayesian Information Criterion (BIC). Phylogenetic tree was visualized and annotated using iTOL [14].

**
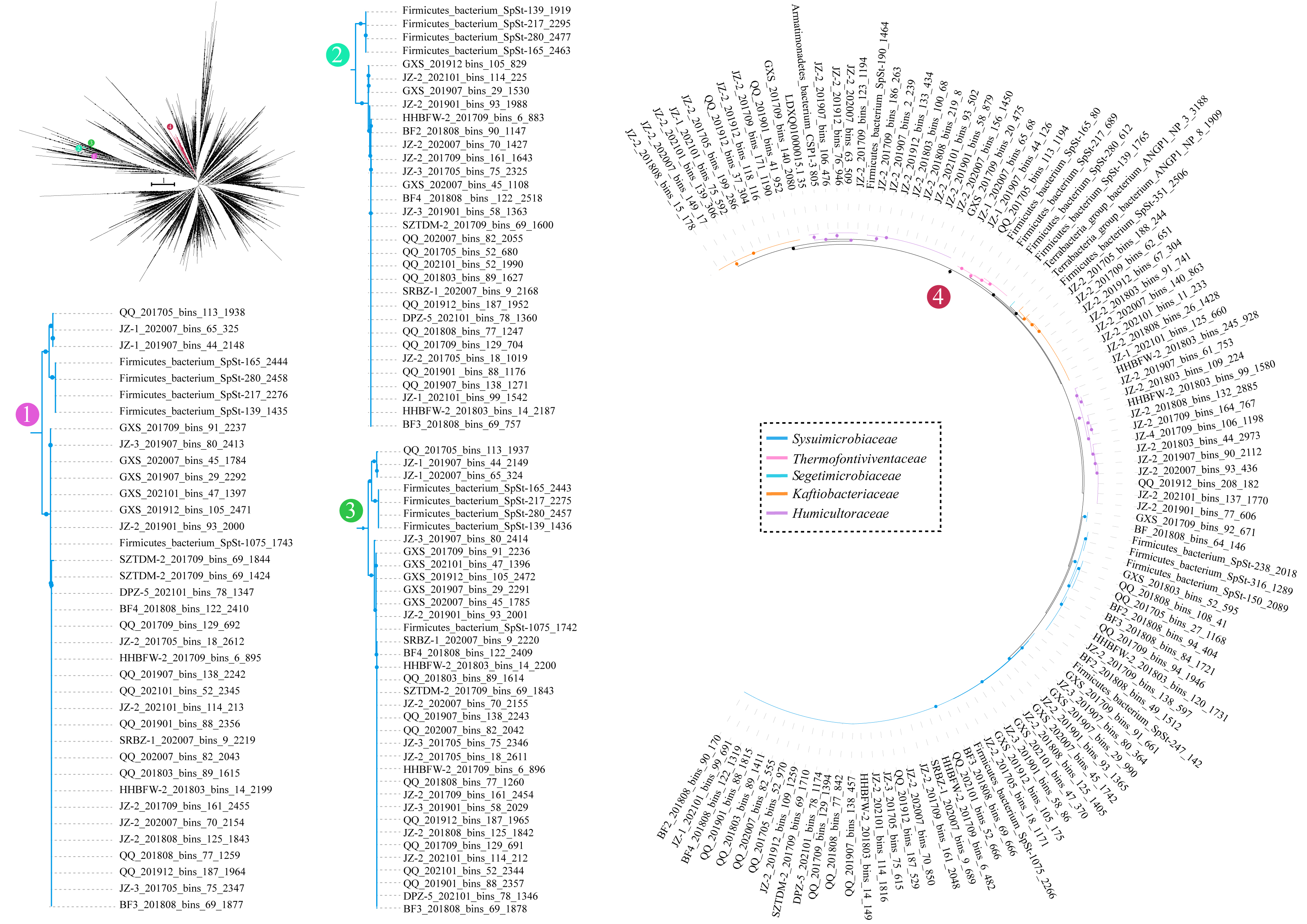
**

**Supplementary Fig. S15. Phylogenetic tree of GcvH protein sequences.** The GcvH sequences were aligned using MUSCLE5 [11], and divergent regions were eliminated using TrimAL [12]. The IQ-Tree was used for phylogenetic inference [13], and the best model LG+R10 was well supported by Bayesian Information Criterion (BIC). Phylogenetic tree was visualized and annotated using iTOL [14].

**
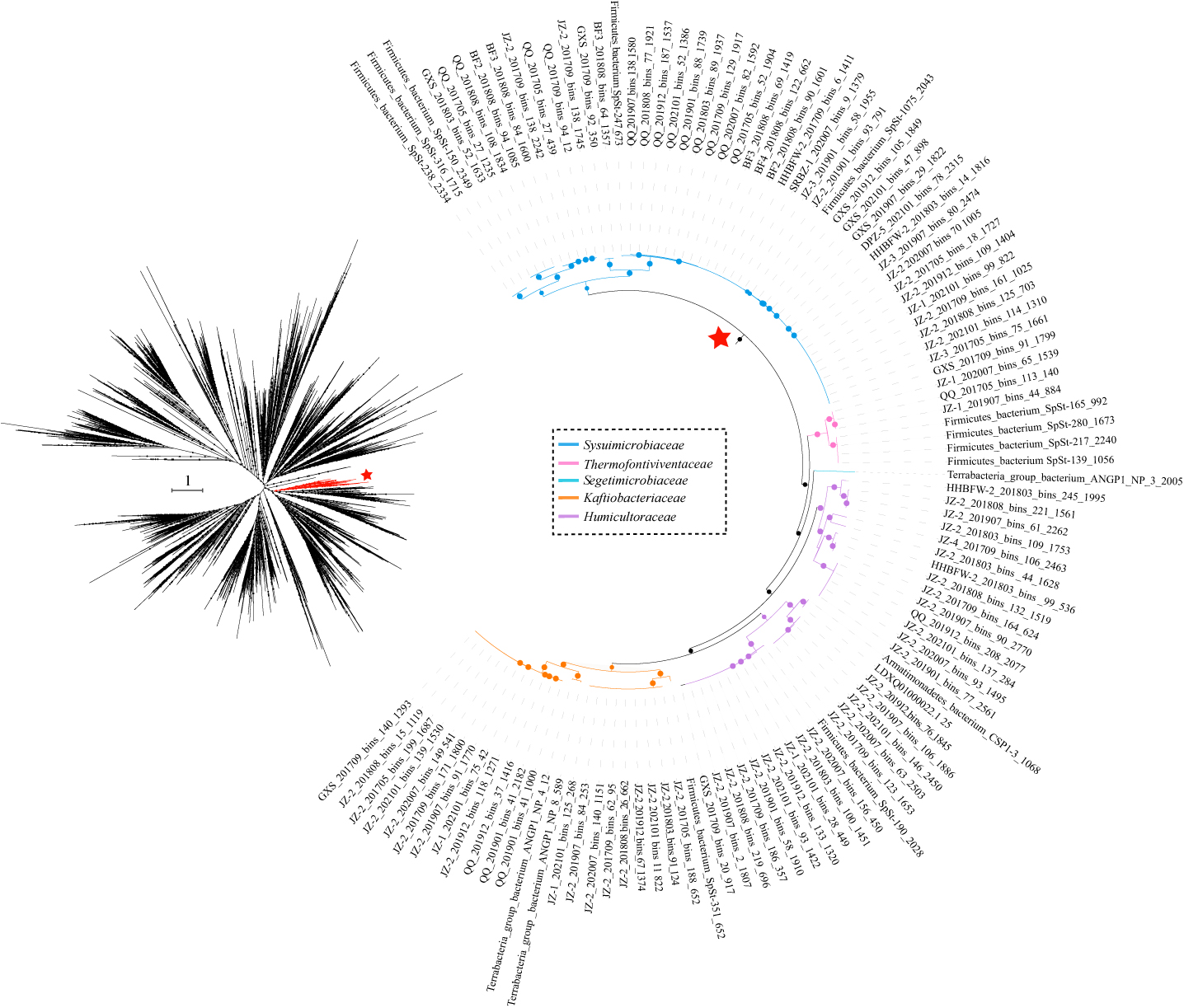
**

**Supplementary Fig. S16. Phylogenetic tree of PdhD protein sequences.** The PdhD sequences were aligned using MUSCLE5 [11], and divergent regions were eliminated using TrimAL [12]. The IQ-Tree was used for phylogenetic inference [13], and the best model LG+R10 was well supported by Bayesian Information Criterion (BIC). Phylogenetic tree was visualized and annotated using iTOL [14].

**
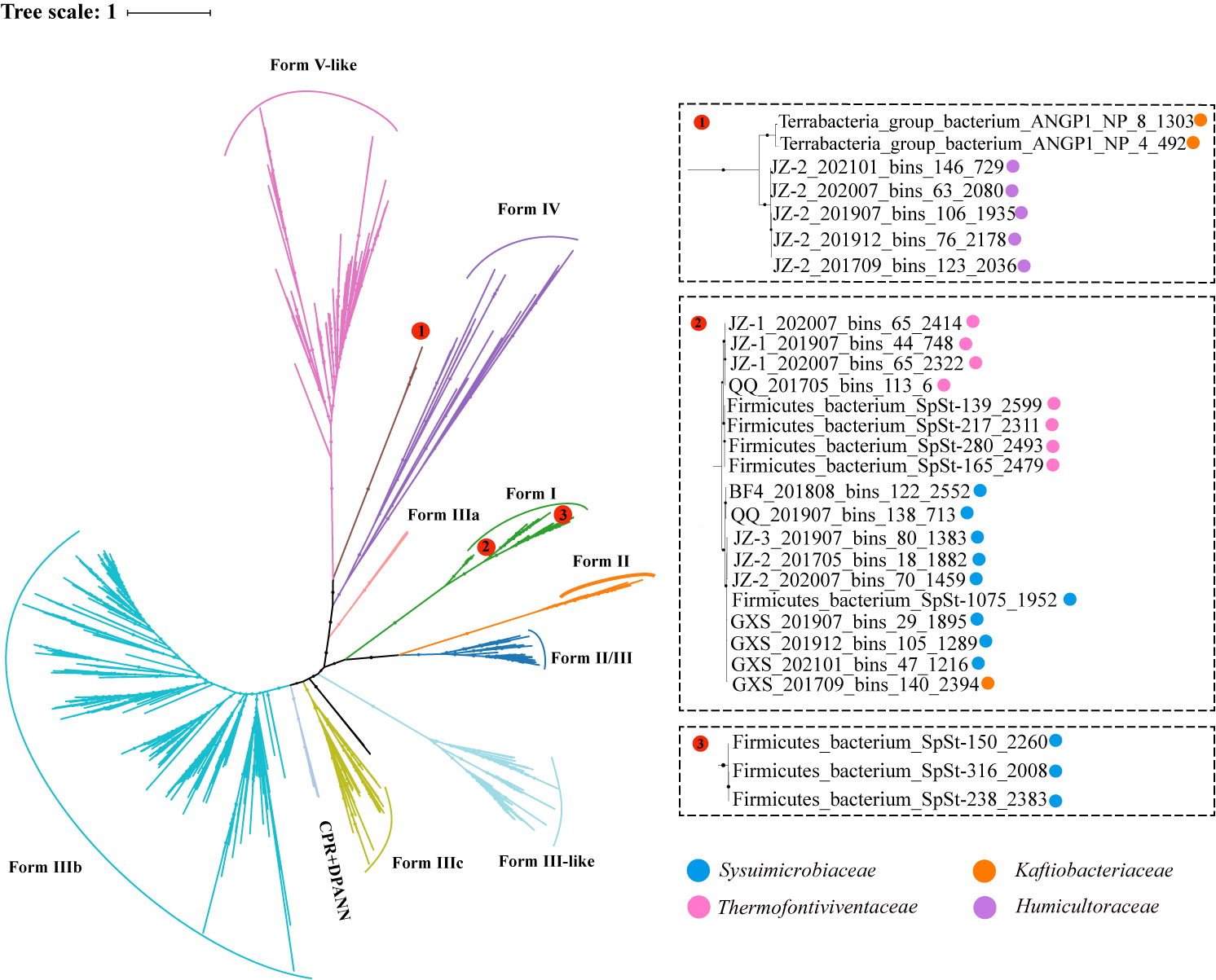
**

**Supplementary Fig. S17. The phylogenetic tree of RuBisCO large subunit.** Reference sequences of ribulose-1,5-bisphosphate carboxylase/oxygenase (RuBisCO) large subunit were obtained from previous study [18]. All sequences were aligned using MUSCLE5 [11] with 100 iterations, and poorly aligned regions were removed using TrimAL [12]. The phylogenetic tree was inferred by IQ-Tree [13], and LG+F+R10 was chosen as the best-fit model according to BIC. Bootstrap values ≥ 70 was shown in solid dots.

**
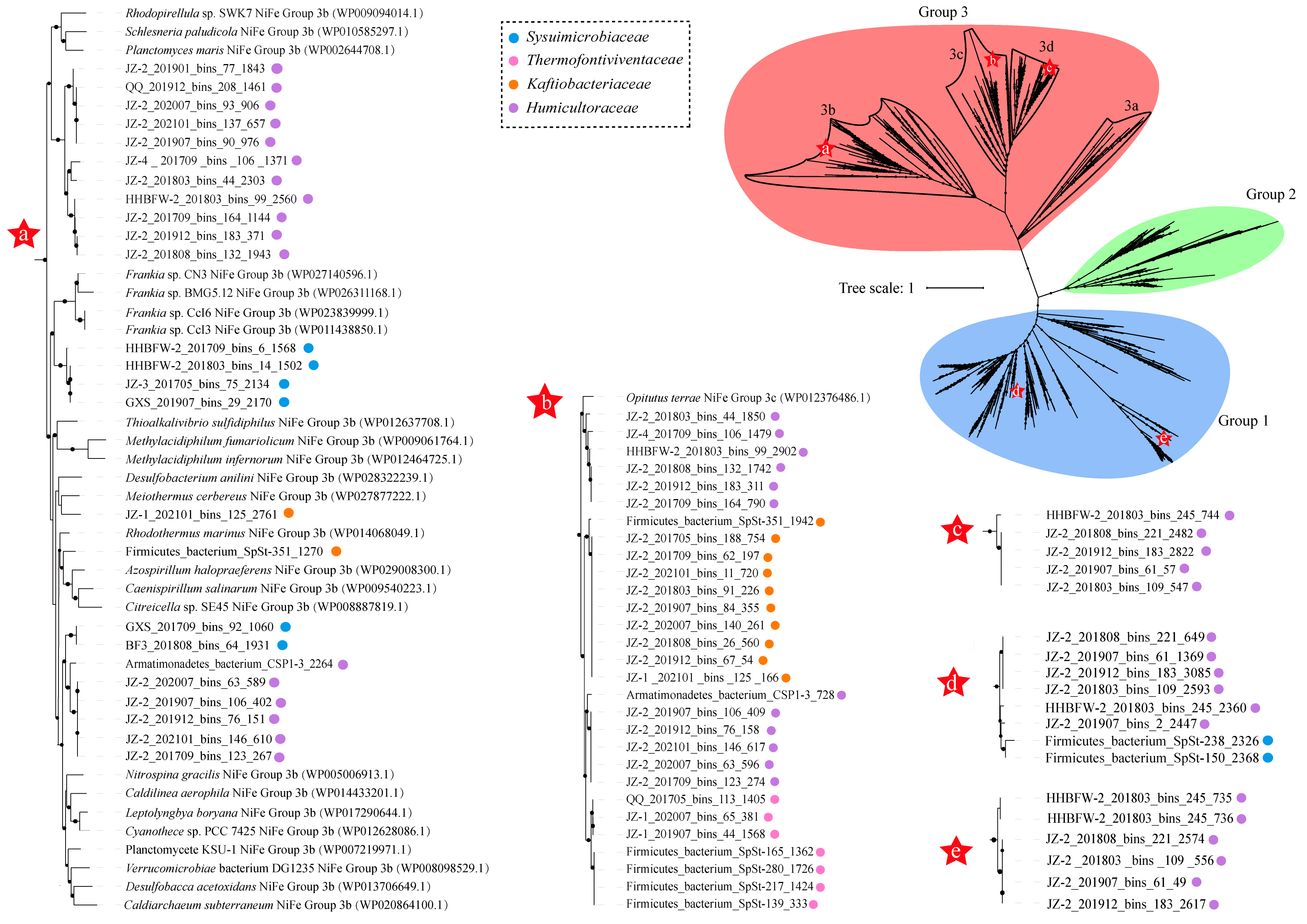
**

**Supplementary Fig. S18. Phylogenetic tree of** **groups 1, 2 and 3 [NiFe] hydrogenases catalytic subunits.** The reference dataset of hydrogenases was selected from one previous study ^[19]^. Alignments were generated using MUSCLE5 [11] with 100 iterations and divergent regions were eliminated using TrimAL [12]. The phylogenomic tree was generated using IQ-TREE [13] (v1.6.10; -alrt 1000 -bb 1000 -nt AUTO). The best-fit model (WAG+F+R10) determined by ModelFinder is well supported by Bayesian Information Criterion (BIC). Bootstrap values > 70% were shown on nodes. Scale bar indicates substitutions per site. The target protein sequences were annotated against the HydDB [20] and Pfam [21] databases.

**
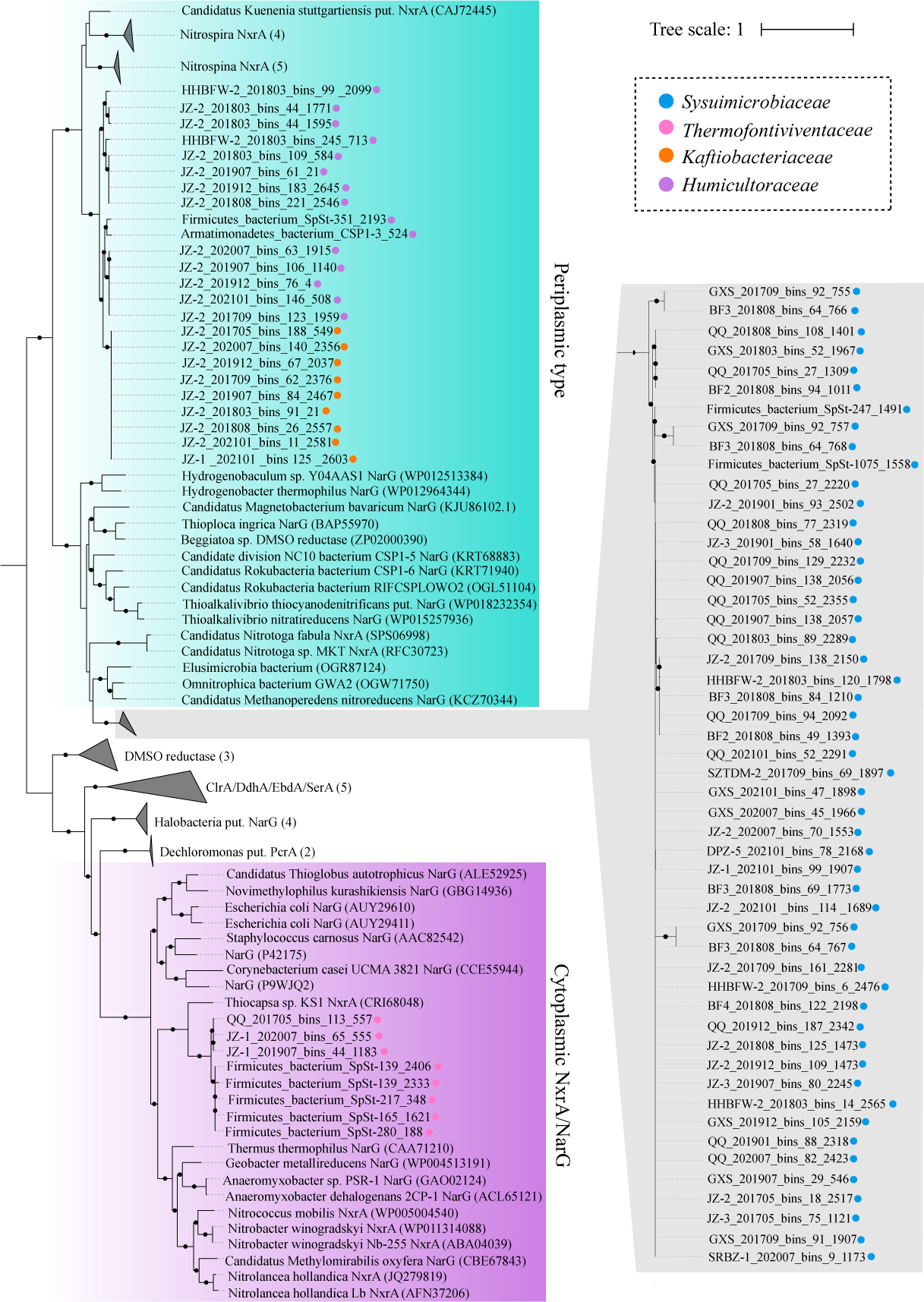
**

**Supplementary Fig. S19. The phylogenetic tree of NxrA/NarG.** All sequences were aligned using MUSCLE5 [11] with 100 iterations, and poorly aligned regions were removed using TrimAL [12]. The phylogenetic tree was inferred by IQ-Tree [13], and LG+F+R4 was chosen as the best-fit model according to BIC. Bootstrap values ≥ 50 was shown in solid dots.


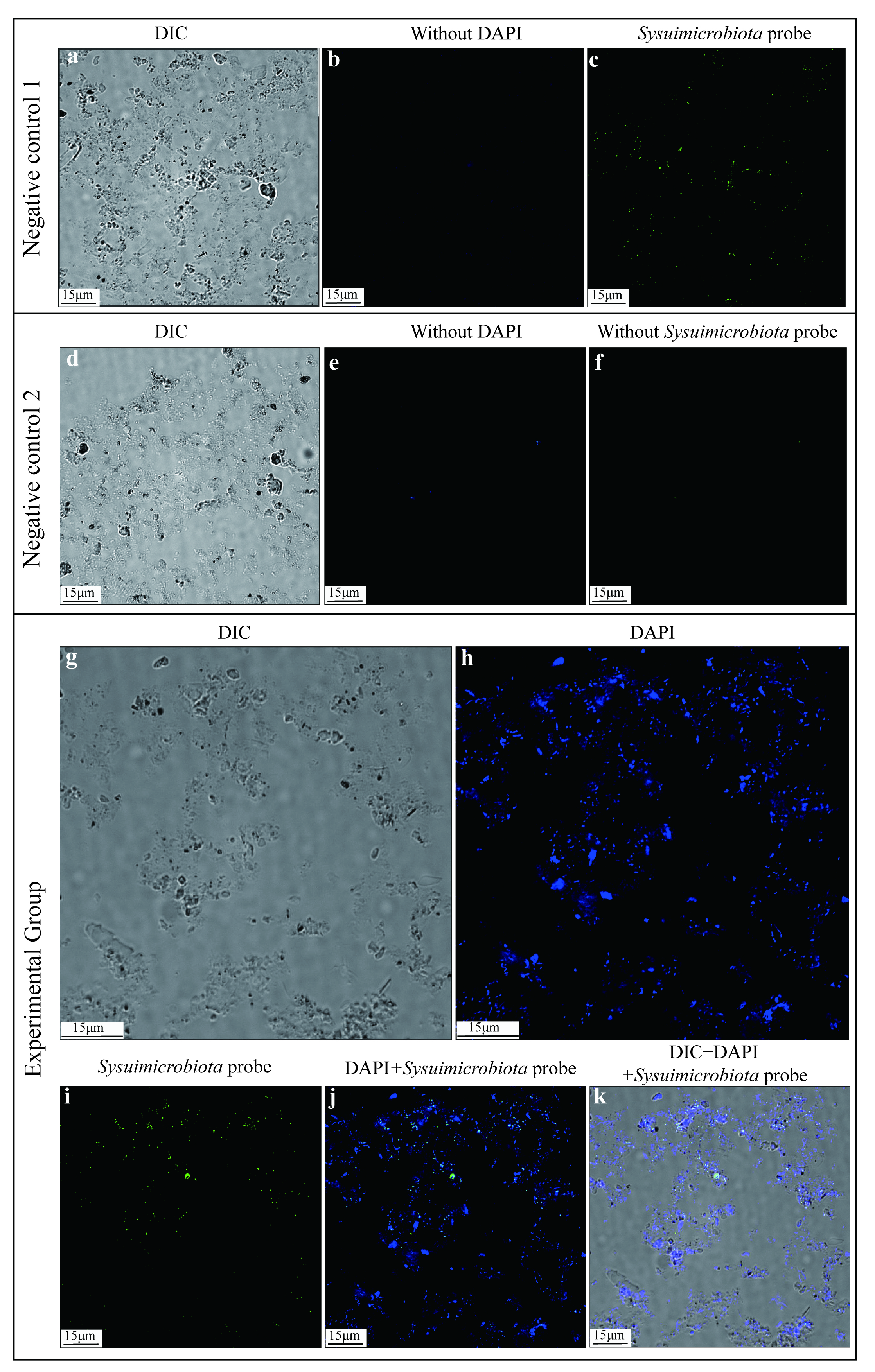


**Supplementary Fig. S20.** **Fluorescence in situ hybridization (FISH) images of *Sysuimicrobiota* in enrichment culture**. a-c: FISH negative control 1, enrichment cells were hybridized by *Sysuimicrobiota* probe but without DAPI staining (a, DIC (Differential Interference Contrast) image; b, image without DAPI staining; c, image with *Sysuimicrobiota* probe); d-f: FISH negative control 2, enrichment cells without DAPI staining and *Sysuimicrobiota* probe hybridization (d, DIC image; e, image without DAPI staining; f, image without *Sysuimicrobiota* probe). g-k: Images with *Sysuimicrobiota* probe or/and DAPI staining. (g, DIC image; h, DNA stained with DAPI (blue); i, *Sysuimicrobiota* cells visualized with FITC-labeled oligonucleotide probe (green); j, DNA stained with DAPI (blue) and *Sysuimicrobiota* cells visualized with FITC-labeled oligonucleotide probe (green); k, merged image of DIC and fluorescence).


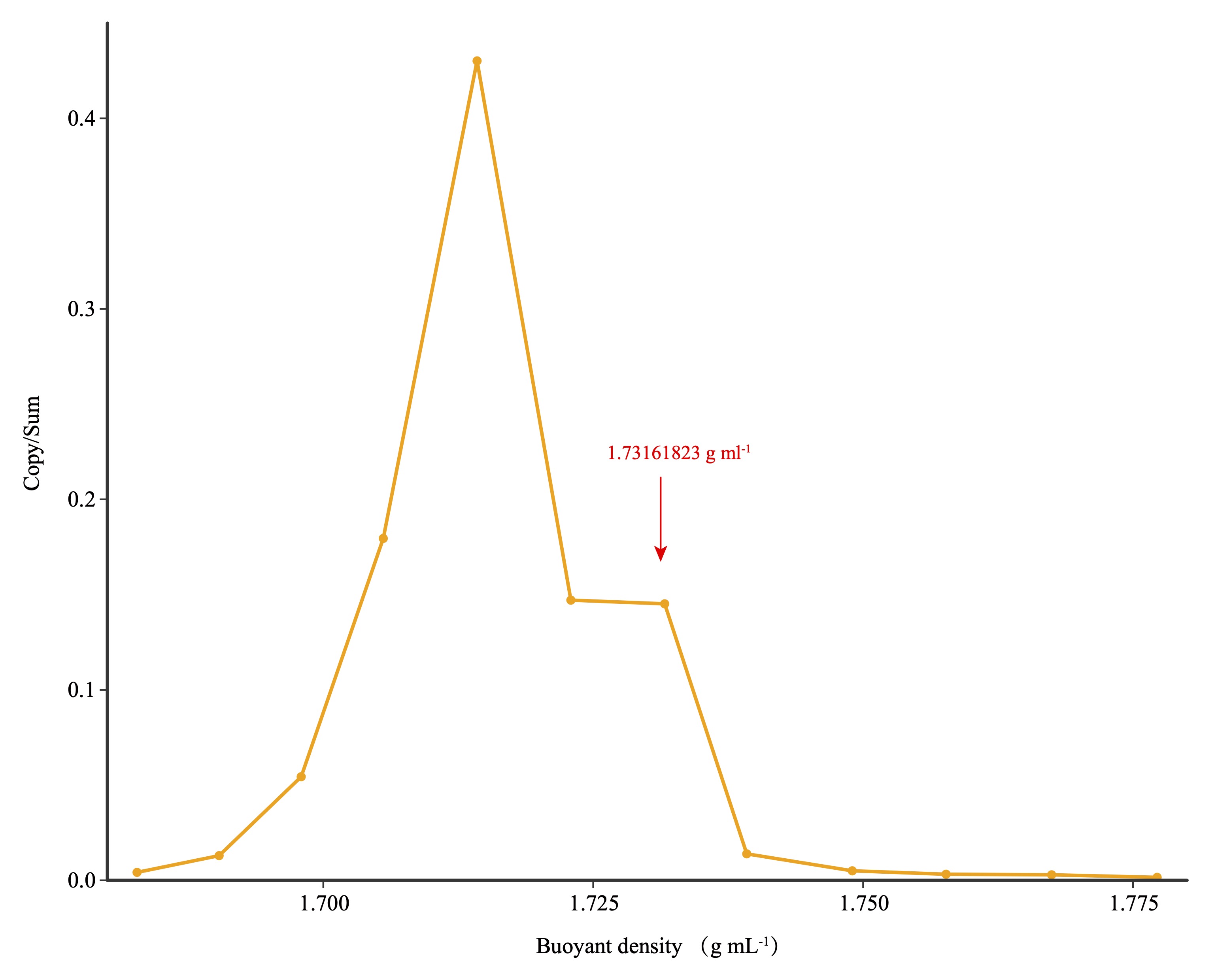


**Supplementary Fig. S21. Quantification of total bacterial 16S rRNA genes across density fractions.** Fractions were collected from gradients from ^13^C-DNA treatment, and quantities of 16S rRNA genes were assessed by qPCR.

**
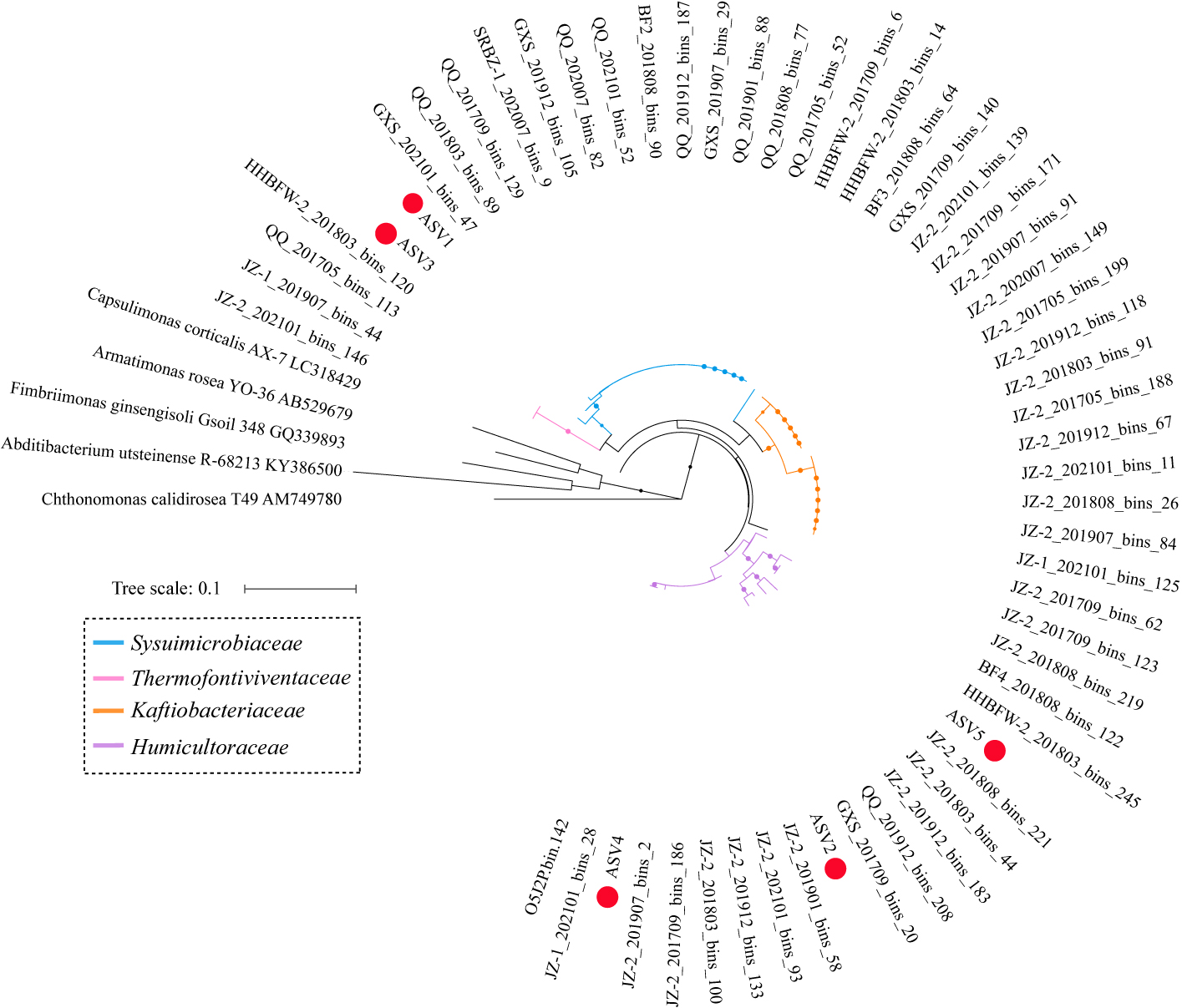
**

**Supplementary Fig. S22. The phylogenetic tree based on 16S rRNA gene sequences of *Sysuimicrobiota* ASVs in the heavy DNA fractions of the ^13^C treatment and *Sysuimicrobiota* MAGs in this study.** Sequence data were aligned using CLUSTAL_X version 1.83 [22]. Phylogenetic trees were generated using MEGA version X [23]. Bootstrap values of above 70 % are indicated at the branch points. Bar, 0.005, represents substitutions per nucleotide position.

**
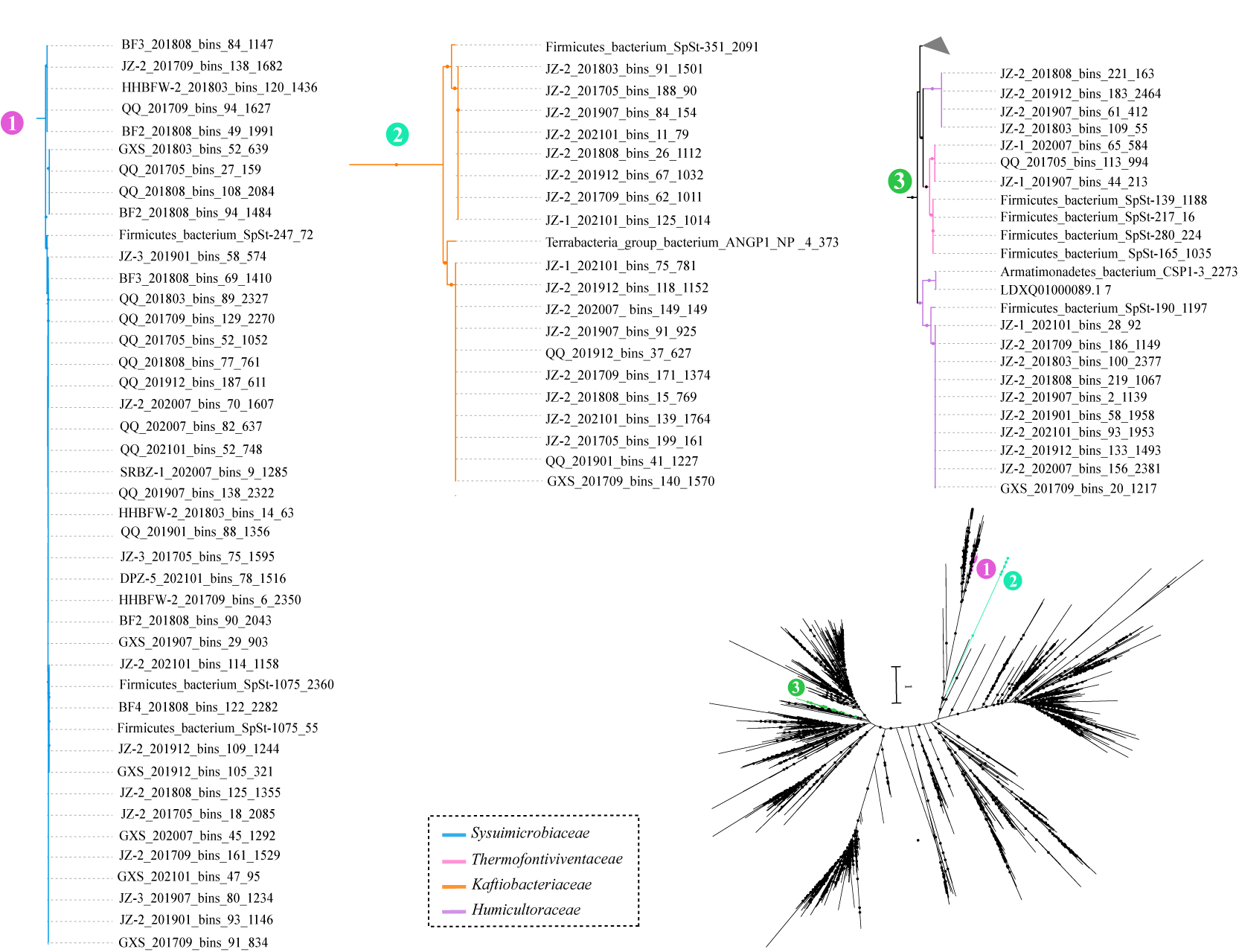
**

**Supplementary Fig. S23. The phylogenetic tree of NirK.** All sequences were aligned using MUSCLE5 [11] with 100 iterations, and poorly aligned regions were removed using TrimAL [12]. The phylogenetic tree was inferred by IQ-Tree [13], and WAG+R10 was chosen as the best-fit model according to BIC. Bootstrap values ≥ 70 was shown in solid dots.


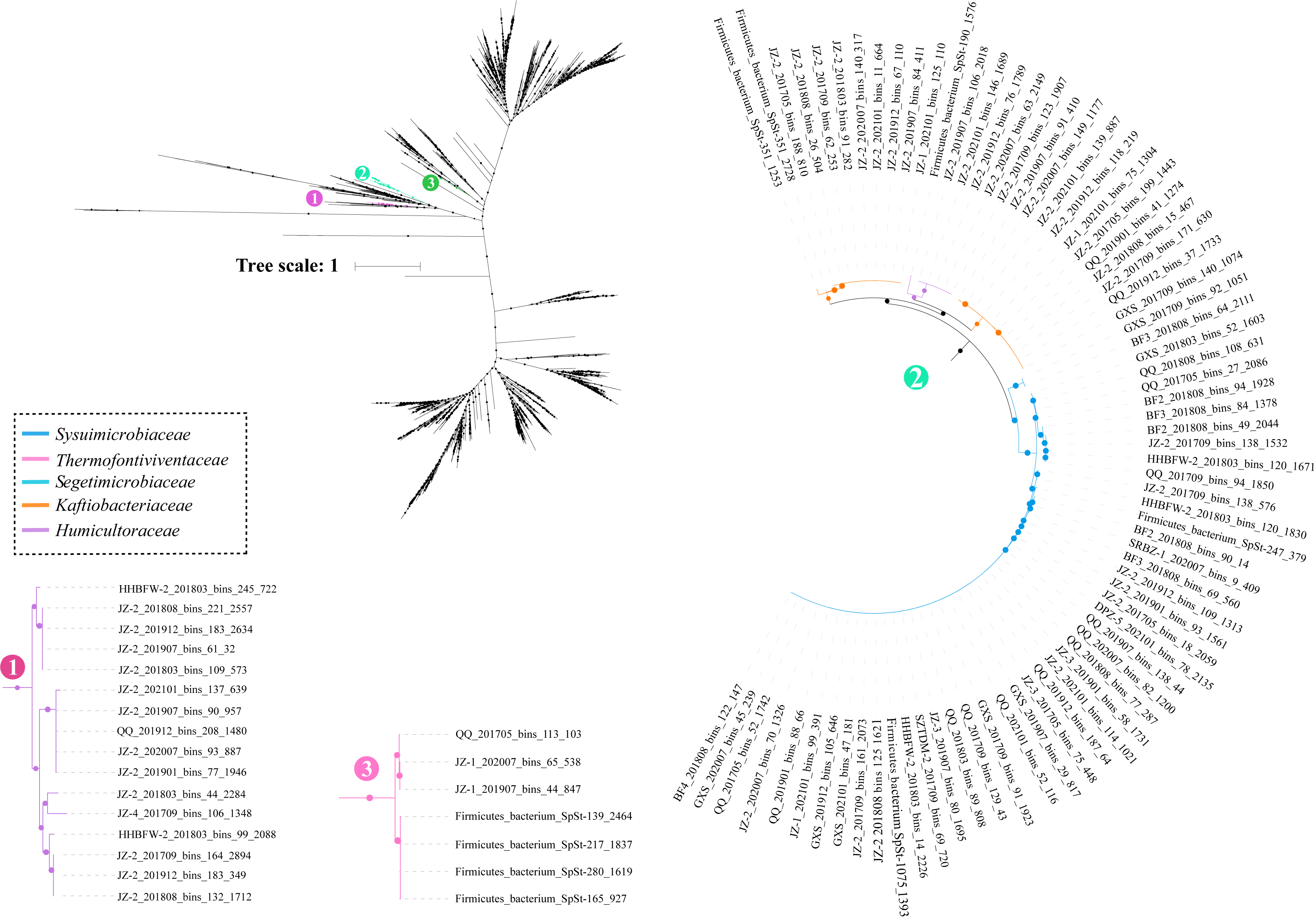


**Supplementary Fig. S24. The phylogenetic tree of NosZ.** All sequences were aligned using MUSCLE5 [11] with 100 iterations, and poorly aligned regions were removed using TrimAL [12]. The phylogenetic tree was inferred by IQ-Tree [13], and LG+F+R10 was chosen as the best-fit model according to BIC. Bootstrap values ≥ 70 was shown in solid dots.

**
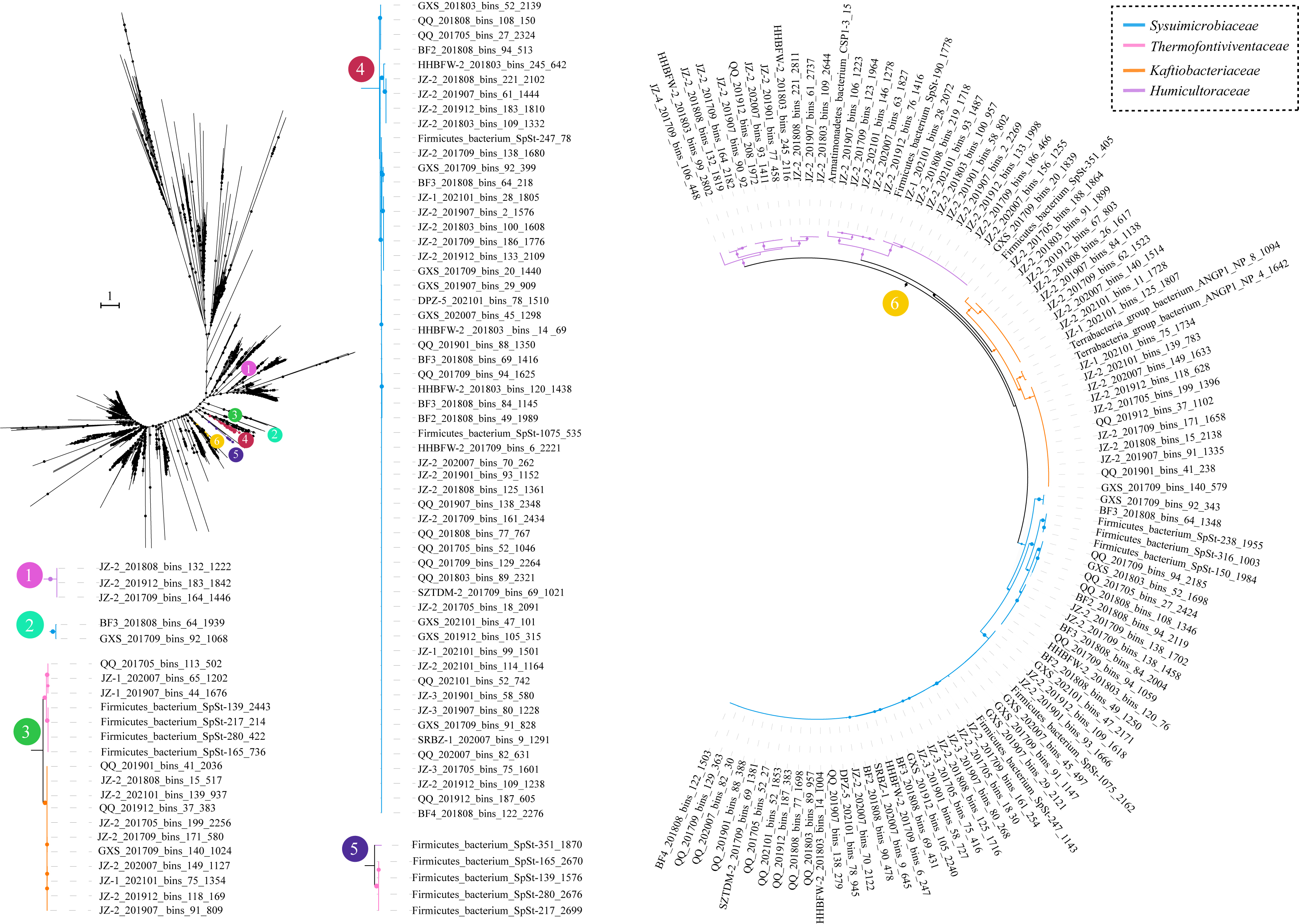
**

**Supplementary Fig. S25. The phylogenetic tree of Sqr.** All sequences were aligned using MUSCLE5 [11] with 100 iterations, and poorly aligned regions were removed using TrimAL [12]. The phylogenetic tree was inferred by IQ-Tree [13], and LG+R10 was chosen as the best-fit model according to AIC and BIC. Bootstrap values ≥ 70 was shown in solid dots.

**REFERENCES**

1. Wang S, Zhu G, Peng Y *et al.* Anammox bacterial abundance, activity, and contribution in riparian sediments of the Pearl River estuary. *Environ Sci Technol*. 2012; **46**: 8834-8842.

2. Hou L, Zheng Y, Liu M *et al.* Anaerobic ammonium oxidation (anammox) bacterial diversity, abundance, and activity in marsh sediments of the Yangtze Estuary. *J Geophys Res: Biogeosci*. 2013; **118**: 1237-1246.

3. Chesnin L, Yien CH. Turbidimetric determination of available sulphates. *Proc Soil Sci Soc Am* 1951; **15:** 149-151.

4. Caporaso JG, Lauber CL, Walters WA *et al.* Global patterns of 16S rRNA diversity at a depth of millions of sequences per sample. *Proc Natl Acad Sci U S A*. 2011; **108**: 4516-4522.

5. Daims H, Lucker S, Wagner M. A new perspective on microbes formerly known as nitrite-oxidizing bacteria. *Trends Microbiol*. 2016; **24**: 699-712.

6. Strous M, Pelletier E, Mangenot S *et al.* Deciphering the evolution and metabolism of an anammox bacterium from a community genome. *Nature*. 2006; **440**: 790-794.

7. Hein S, Simon J. Bacterial nitrous oxide respiration: electron transport chains and copper transfer reactions. *Adv Microb Physiol*. 2019; **75**: 137-175.

8. Kuypers MMM, Marchant HK, Kartal B. The microbial nitrogen-cycling network. *Nat Rev Microbiol*. 2018; **16**: 263-276.

9. Pruesse E, Quast C, Knittel K *et al.* SILVA: a comprehensive online resource for quality checked and aligned ribosomal RNA sequence data compatible with ARB. *Nucleic Acids Res*. 2007; **35**: 7188-7196.

10. Ludwig W, Strunk O, Westram R *et al.* ARB: a software environment for sequence data. *Nucleic Acids Res*. 2004; **32**: 1363-1371.

11. Edgar RC. Muscle5: High-accuracy alignment ensembles enable unbiased assessments of sequence homology and phylogeny. *Nat Commun*. 2022; **13**: 6968.

12. Capella-Gutiérrez S, Silla-Martínez JM, Gabaldón T. trimAl: a tool for automated alignment trimming in large-scale phylogenetic analyses. *Bioinformatics*. 2009; **25**: 1972-1973.

13. Nguyen LT, Schmidt HA, von Haeseler A *et al.* IQ-TREE: a fast and effective stochastic algorithm for estimating maximum-likelihood phylogenies. *Mol Biol Evol*. 2015; **32**: 268-274.

14. Letunic I, Bork P. Interactive Tree Of Life (iTOL) v4: recent updates and new developments. *Nucleic Acids Res*. 2019; **47**: W256-W259.

15. Jiao JY, Lian ZH, Li MM *et al.* Comparative genomic analysis of *Thermus* provides insights into the evolutionary history of an incomplete denitrification pathway. *mLife*. 2022; **1**: 198-209.

16. Adam PS, Borrel G, Gribaldo S. Evolutionary history of carbon monoxide dehydrogenase/acetyl-CoA synthase, one of the oldest enzymatic complexes. *Proc Natl Acad Sci U S A*. 2018; **115**: E1166-e1173.

17. Raymann K, Brochier-Armanet C, Gribaldo S. The two-domain tree of life is linked to a new root for the Archaea. *Proc Natl Acad Sci U S A*. 2015; **112**: 6670-6675.

18. Jaffe AL, Castelle CJ, Dupont CL *et al.* Lateral gene transfer shapes the distribution of RuBisCO among Candidate Phyla Radiation Bacteria and DPANN Archaea. *Mol Biol Evol*. 2019; **36**: 435-446.

19. Greening C, Biswas A, Carere CR *et al.* Genomic and metagenomic surveys of hydrogenase distribution indicate H_2_ is a widely utilised energy source for microbial growth and survival. *ISME J*. 2016; **10**: 761-777.

20. Søndergaard D, Pedersen CN, Greening C. HydDB: a web tool for hydrogenase classification and analysis. *Sci Rep*. 2016; **6**: 1-8.

21. El-Gebali S, Mistry J, Bateman A *et al.* The Pfam protein families database in 2019. *Nucleic Acids Res*. 2019; **47**: D427-D432.

22. Thompson JD, Gibson TJ, Plewniak F *et al.* The CLUSTAL_X windows interface: flexible strategies for multiple sequence alignment aided by quality analysis tools. *Nucleic Acids Res*. 1997; **25**: 4876-4882.

23. Kumar S, Stecher G, Li M *et al.* MEGA X: Molecular evolutionary genetics analysis across computing platforms. *Mol Biol Evol*. 2018; **35**: 1547-1549.
